# Independent effects of adiposity measures on risk of atrial fibrillation in men and women: a study of 0.5 million individuals

**DOI:** 10.1093/ije/dyab184

**Published:** 2021-09-25

**Authors:** C Fielder Camm, Ben Lacey, M Sofia Massa, Adam Von Ende, Parag Gajendragadkar, Alexander Stiby, Elsa Valdes-Marquez, Sarah Lewington, Rohan Wijesurendra, Sarah Parish, Barbara Casadei, Jemma C Hopewell

**Affiliations:** Nuffield Department of Population Health, University of Oxford, Oxford, UK; Nuffield Department of Population Health, University of Oxford, Oxford, UK; Nuffield Department of Population Health, University of Oxford, Oxford, UK; Nuffield Department of Population Health, University of Oxford, Oxford, UK; Nuffield Department of Population Health, University of Oxford, Oxford, UK; Nuffield Department of Population Health, University of Oxford, Oxford, UK; Nuffield Department of Population Health, University of Oxford, Oxford, UK; Nuffield Department of Population Health, University of Oxford, Oxford, UK; MRC Population Health Research Unit, Nuffield Department of Population Health, University of Oxford, Oxford, UK; Nuffield Department of Population Health, University of Oxford, Oxford, UK; Division of Cardiovascular Medicine, Radcliffe Department of Medicine, University of Oxford, Oxford, UK; Nuffield Department of Population Health, University of Oxford, Oxford, UK; MRC Population Health Research Unit, Nuffield Department of Population Health, University of Oxford, Oxford, UK; Division of Cardiovascular Medicine, Radcliffe Department of Medicine, University of Oxford, Oxford, UK; Nuffield Department of Population Health, University of Oxford, Oxford, UK

**Keywords:** Atrial fibrillation, obesity, adiposity, UK Biobank, sex

## Abstract

**Background:**

Atrial fibrillation (AF) has a higher prevalence in men than in women and is associated with measures of adiposity and lean mass (LM). However, it remains uncertain whether the risks of AF associated with these measures vary by sex.

**Methods:**

Among 477 904 UK Biobank participants aged 40–69 without prior AF, 23 134 incident AF cases were identified (14 400 men, 8734 women; median follow-up 11.1 years). Cox proportional hazards models were used to estimate the covariate adjusted hazard ratios (HRs) describing the association of AF with weight, measures of adiposity [fat mass (FM), waist circumference (WC)] and LM, and their independent relevance, by sex.

**Results:**

Weight and WC were independently associated with risk of AF [HR: 1.25 (1.23–1.27) per 10 kg, HR: 1.11 (1.09–1.14) per 10 cm, respectively], with comparable effects in both sexes. The association with weight was principally driven by LM, which, per 5 kg, conferred double the risk of AF compared with FM when mutually adjusted [HR: 1.20 (1.19–1.21), HR: 1.10 (1.09–1.11), respectively]; however, the effect of LM was weaker in men than in women (*p*-interaction = 4.3 x 10^−9^). Comparing the relative effects of LM, FM and WC identified different patterns within each sex; LM was the strongest predictor for both, whereas WC was stronger than FM in men but not in women.

**Conclusions:**

LM and FM (as constituents of weight) and WC are risk factors for AF. However, the independent relevance of general adiposity for AF was more limited in men than in women. The relevance of both WC and LM suggests a potentially important role for visceral adiposity and muscle mass in AF development.

Key Messages

- Lean mass (LM), fat mass (FM) and waist circumference are independent risk factors for atrial fibrillation (AF) in both men and women.
- LM confers a 1.5- and 2-fold greater risk of AF vs FM per kg in men and women, respectively.
- The relative effects of LM and adiposity measures on risk of AF suggest that the relevance of body composition, particularly in the context of AF-focused weight-loss programmes, merits further investigation.

## Introduction

Atrial fibrillation (AF) is a common cardiac arrhythmia^1^ and is a major cause of death and disability, primarily through its impact on risk of cardio-embolic stroke and heart failure.^2^ The age-adjusted prevalence of AF is lower in women than in men.^3^ Additionally, there are sex-based differences in the risk of AF-related complications, with a higher risk of stroke/thromboembolism,^4^ a higher symptom burden, but a lower risk of death in women vs men.^5^

Worldwide, an ∼30% increase in the incidence of AF between 1990 and 2010 has been attributed, in part, to the rising levels of obesity.^3^^,^^6^ Observational studies have shown positive associations between risk of AF and measures of general adiposity [such as body mass index (BMI)], central adiposity [such as waist circumference (WC)] and body lean mass (LM).^7–9^ Conversely, weight loss has been associated with a lower AF burden and maintenance of sinus rhythm.^10^ Genetic studies also support a causal role for both LM and body-fat mass (FM) in the development of AF.^11^

Body fat distribution differs between sexes,^12–14^ with men having lower FM and higher LM (per unit BMI) compared with women.^15^ Men also have higher levels of visceral fat compared with women.^14^^,^^16^ Visceral adiposity has been shown to be correlated with WC in small studies^17^ and is associated with risk of cardiovascular disease, independently of general adiposity.^18^ Visceral adipose tissue is also positively correlated with levels of pro-inflammatory cytokines, such as interleukin-6, which have been associated with risk of AF.^19^^,^^20^ LM has previously been correlated with left ventricular mass and left atrial size, which may underlie some of the association between LM and risk of AF.^21^^,^^22^ Consequently, differences in LM and fat distribution, through their potential impact on biological pathways, may contribute to the sex-related differences in the prevalence of AF and provide important insights into the pathogenesis of AF and its complications.

Reliable assessments of sex-specific effects of general adiposity, body fat distribution and LM on the risk of AF have been limited by data availability.^23–26^ Large-scale prospective cohorts now offer the possibility to obtain more robust assessments. Here, we aimed to compare the independent effects of general adiposity, central adiposity and LM on the risk of AF in 215 196 men and 262 708 women in UK Biobank (UKB).

## Methods

### Study design and participants

Details of the UKB and data collection methods have been reported previously.^27^ In brief, the UKB is a prospective cohort of 502 493 adults recruited between 2006 and 2010. At recruitment, men and women, aged 40–69 years, underwent a comprehensive interview detailing socio-demographic, lifestyle, environmental and other health-related factors, and completed a range of physical measures, including weight, height and WC.^28^ Bio-impedance was used to provide data on whole-body FM and LM, and was measured using a Tanita BC418MA body composition analyser. See Supplementary Material (available as Supplementary data at *IJE* online) for further details on relevant data availability and collection methods .

### AF and other health outcomes

Incident cases of AF (and comorbid diseases) were identified using hospital inpatient admissions data from hospital episode statistics (HES) (based on any reason for admission; additional details shown in Supplementary Table S1, available as Supplementary data at *IJE* online) and death registry data following recruitment. Health outcomes prior to recruitment were identified from HES records and self-reported information.

### Statistical analyses

A total of 477 904 participants were included in the primary analyses, after excluding 17 635 participants with missing or extreme anthropometric values or predefined covariates^29^^,^^30^ and 6954 with prevalent AF (Supplementary Figure S1, available as Supplementary data at *IJE* online). Cox proportional hazards regression models were used to estimate hazard ratios (HRs) describing the associations between incident AF and each anthropometric measure: BMI, WC, weight, FM and LM. Results were adjusted for the following potentially confounding covariates: age at risk (in 5-year age groups), sex, ethnicity, Townsend deprivation index,^31^ alcohol intake and smoking (never, former, current).

In order to explore the shape of the associations, anthropometric measures were classified, separately in men and in women, into fifths, with the top group being further subdivided into two (corresponding to the top two tenths of the distribution), thereby keeping case numbers similar in each group. The relative independence of different anthropometric traits was assessed by additional adjustment for these grouped variables.

In figures, estimates for each group are shown relative to a defined reference group (the second fifth was the selected reference group). Group-specific variances were used to calculate 95% confidence intervals (CIs) to enable comparisons between any two groups (rather than with the arbitrary reference group).^32^

Estimated HRs are based on using the anthropometric measure as a continuous variable throughout the text except where indicated. χ^2^ values are derived from likelihood ratio statistics. Details of sensitivity analyses conducted are provided in the Supplementary Methods (available as Supplementary data at *IJE* online). All statistical analyses were undertaken using SAS (Version 9.3).

## Results

### Baseline characteristics

Of the 477 904 participants included in the analyses, 55% were women. At baseline, participants had a mean age of 56.4 years [standard deviation (SD) 8.1], which was similar between sexes (Table  1). Participants were primarily of White ethnicity (95%) and about half (46%) were from the least disadvantaged fifth of the UK population (as defined by Townsend deprivation index scores). About 10% of both sexes were current smokers and most were current alcohol drinkers (92%). There was a higher prevalence of coronary heart disease (6.5% vs 2.2%) and diabetes (6.5% vs 3.3%) in men vs women.

**Table 1:** Baseline characteristics of the 477 904 participants in the main analysis

	Men	Women	Total
	(*n* = 215 196)	(*n* = 262 708)	(*n* = 477 904)
Demographic and lifestyle factors			
Age [mean (SD)]	56.6 (8.2)	56.3 (8.0)	56.4 (8.1)
White [*n* (%)]	203 737 (94.7)	248 797 (94.7)	452 534 (94.7)
Current smoker [*n* (%)]	26 906 (12.5)	23 469 (8.9)	50 375 (10.5)
Current alcohol drinker [*n* (%)]	202 093 (93.9)	238 354 (90.7)	440 447 (92.2)
Townsend deprivation index^a^ [mean (SD)]	−1.3 (3.1)	−1.4 (3.0)	−1.3 (3.1)
Anthropometric measures [mean (SD)]			
Height (cm)	175.6 (6.8)	162.5 (6.3)	168.4 (9.3)
Weight (kg)	85.8 (13.9)	71.3 (13.7)	77.8 (15.6)
Body mass index (kg/m^2^)	27.8 (4.1)	27.0 (5.0)	27.4 (4.7)
Waist circumference (cm)	96.8 (11.0)	84.5 (12.3)	90.1 (13.2)
Hip circumference (cm)	103.3 (7.3)	103.2 (10.1)	103.3 (9.0)
Waist–hip ratio	0.9 (0.1)	0.8 (0.1)	0.9 (0.1)
Bio-impedance measures [mean (SD)]			
Lean mass (kg)	63.6 (7.7)	44.5 (5.0)	53.1 (11.4)
Fat mass (kg)	22.2 (8.0)	26.8 (9.8)	24.7 (9.3)
Prior disease [*n* (%)]			
Congestive cardiac failure	1099 (0.5)	469 (0.2)	1568 (0.3)
Coronary heart disease	13 924 (6.5)	5891 (2.2)	19 815 (4.1)
Diabetes	13 955 (6.5)	8707 (3.3)	22 662 (4.7)
Hypertension	88 719 (41.2)	82 898 (31.6)	171 617 (35.9)
Sleep apnoea	2272 (1.1)	735 (0.3)	3007 (0.6)
Stroke	3375 (1.6)	2444 (0.9)	5819 (1.2)
Valvular heart disease	1834 (0.9)	2375 (0.9)	4209 (0.9)

People with missing or out-of-range anthropometric measures, missing covariates or prior atrial fibrillation at baseline were excluded.

a Area-level measure of material deprivation [UK range: −5.5 (least deprived) to 14.0 (most deprived)].^31^

On average, BMI, WC and weight were higher in men than in women but had similar variance (Table  1). As expected, LM was higher in men (mean 63.6 kg, SD 7.7) than in women (44.5 kg, SD 5.0), whereas FM was higher in women (mean 26.8 kg, SD 9.8) than in men (mean 22.2 kg, SD 8.0; Table  1 and Supplementary Figure S2, available as Supplementary data at *IJE* online). In each sex, FM was strongly correlated with BMI and WC (*r*^2^ = 0.88–0.94 after adjustment for age; Supplementary Tables S2–S4, available as Supplementary data at *IJE* online) and these measures were also correlated with LM (*r*^2^ = 0.60–0.71).

### Incident AF

During a median follow-up of 11.1 years (interquartile range 10.4–11.8), 23 134 incident AF cases were identified (14 400 in men and 8734 in women). A higher incidence of AF in men than in women was observed in all age groups (Supplementary Figure S3, available as Supplementary data at *IJE* online). The mean age at which AF was reported was 69.1 years (SD 6.7) and was similar in both sexes.

### Comparing the effects of BMI, WC and weight on risk of AF in men and women

**Figure 1:**
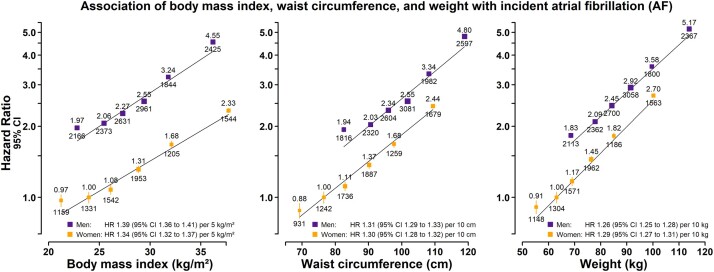
Association of body mass index, waist circumference, and weight with incident atrial fibrillation (AF). Hazard ratios (HRs) were adjusted for age, ethnicity, deprivation, smoking and alcohol. Plotted on a floating absolute scale in men (purple squares) and women (orange squares). Sex-specific regression lines are plotted. Exclusions as per Table 1. For each category, the area of the square is inversely proportional to the variance of the category-specific log risk, which also determines the 95% CI (represented by error bars). The lowest four groups each comprise 20% of the sample, with the highest two groups each comprising 10% of the sample. HRs are shown above each square and the number of AF cases below.

Initially, we investigated the associations between measures of adiposity most commonly used in clinical practice, namely BMI, WC and weight, and the risk of AF. The range of values for these anthropometric measures was comparable between sexes (Figure  1 and Supplementary Figure S2, available as Supplementary data at *IJE* online). Risk of AF was strongly and positively associated with BMI, WC and weight, and the associations were approximately log-linear in both men and women (Figure  1 and Supplementary Figure S4, available as Supplementary data at *IJE* online).

For a given BMI, men were at about double the risk of AF compared with women. BMI was associated with an ∼40% higher risk of AF per 5 kg/m^2^ amongst all participants [HR: 1.37 (95% CI 1.35–1.38)], with a slightly greater effect in men than in women (*p*-interaction = 0.01; Figure  1). However, the risk associated with BMI was more than halved after adjusting for WC [HR: 1.15 (1.12–1.18) per 5 kg/m^2^], after which the effects in both sexes were equivalent (Supplementary Figure S5, available as Supplementary data at *IJE* online).

Similarly, for a given WC, men were at higher risk of AF than women. Overall, WC was associated with a 30% higher risk of AF [HR: 1.30 (1.29–1.32)] per 10 cm (equivalent to a 3.5 to 4 kg/m^2^ change in BMI), with comparable effects in men and women (*p*-interaction = 0.67). This association was independent of BMI, but attenuated after adjustment for weight [HR: 1.11 (1.09–1.14) per 10 cm; Supplementary Figure S6, available as Supplementary data at *IJE* online].

Finally, weight was associated with a 30% higher risk of AF [HR: 1.28 (1.26–1.29)] per 10 kg (equivalent to a 3 to 3.5 kg/m^2^ change in BMI), with comparable associations in both sexes (*p*-interaction = 0.09). In men and women, weight most strongly predicted AF (Supplementary Table S5, available as Supplementary data at *IJE* online). The association between weight and risk of AF was independent of WC [HR: 1.25 (1.23–1.27) per 10 kg], with similar effects in both sexes (Supplementary Figure S5, available as Supplementary data at *IJE* online).

In summary, considered in turn, BMI, WC and weight each predict risk of AF, with weight showing the strongest association in both men and women. The strong and independent association between weight and AF warrants further investigation.

### Comparing the effects of FM and LM on the risk of AF in men and women

**Figure 2:**
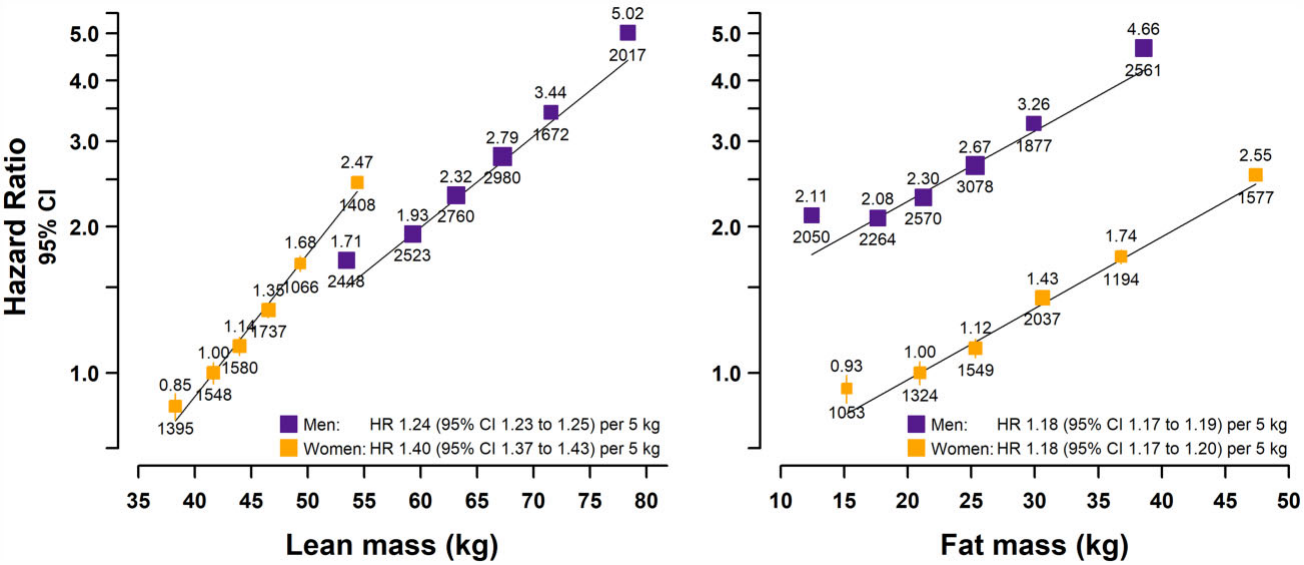
Association of lean mass and fat mass with incident atrial fibrillation (AF). Hazard ratios (HRs) were adjusted for age, ethnicity, deprivation, smoking and alcohol. Plotted on a floating absolute scale in men (purple open squares) and women (orange closed squares). Sex-specific regression lines are plotted. Exclusions as per Table  1. For each category, the area of the square is inversely proportional to the variance of the category-specific log risk, which also determines the 95% CI (represented by error bars). The lowest four groups each comprise 20% of the sample, with the highest two groups each comprising 10% of the sample. HRs are shown above each square and the number of AF cases below.

Weight is the sum of FM and LM, so we assessed the separate effects of FM and LM (derived from bio-impedance measures) on the risk of AF. The distributions for FM were comparable between sexes (Figure  2 and Table  1). However, for LM, men had substantially higher mean levels and variability than women (SD 7.7 vs 4.9 kg) and the mean LM:FM ratio was higher in men than in women (3.21 vs 1.85).

FM and LM were both positively associated with AF and associations were approximately log-linear (Figure  2 and Supplementary Figure S7, available as Supplementary data at *IJE* online). For a given FM, men had an ∼2-fold higher risk of AF than women. In contrast, despite limited overlap in the range of LM values between men and women, women appeared to be at slightly higher risk of AF than men for a given LM. Overall, 5 kg higher FM was associated with a 20% higher risk of AF [HR: 1.18 (1.17–1.19)], with similar effect sizes in both sexes (*p*-interaction = 0.95). LM was associated with a 30% higher AF risk per 5 kg [HR: 1.28 (1.27–1.29)], with a somewhat weaker effect in men [HR: 1.24 (1.23–1.25)] than in women [HR: 1.40 (1.37–1.43)], *p*-interaction = 3 x 10^−^^20^.

**Figure 3:**
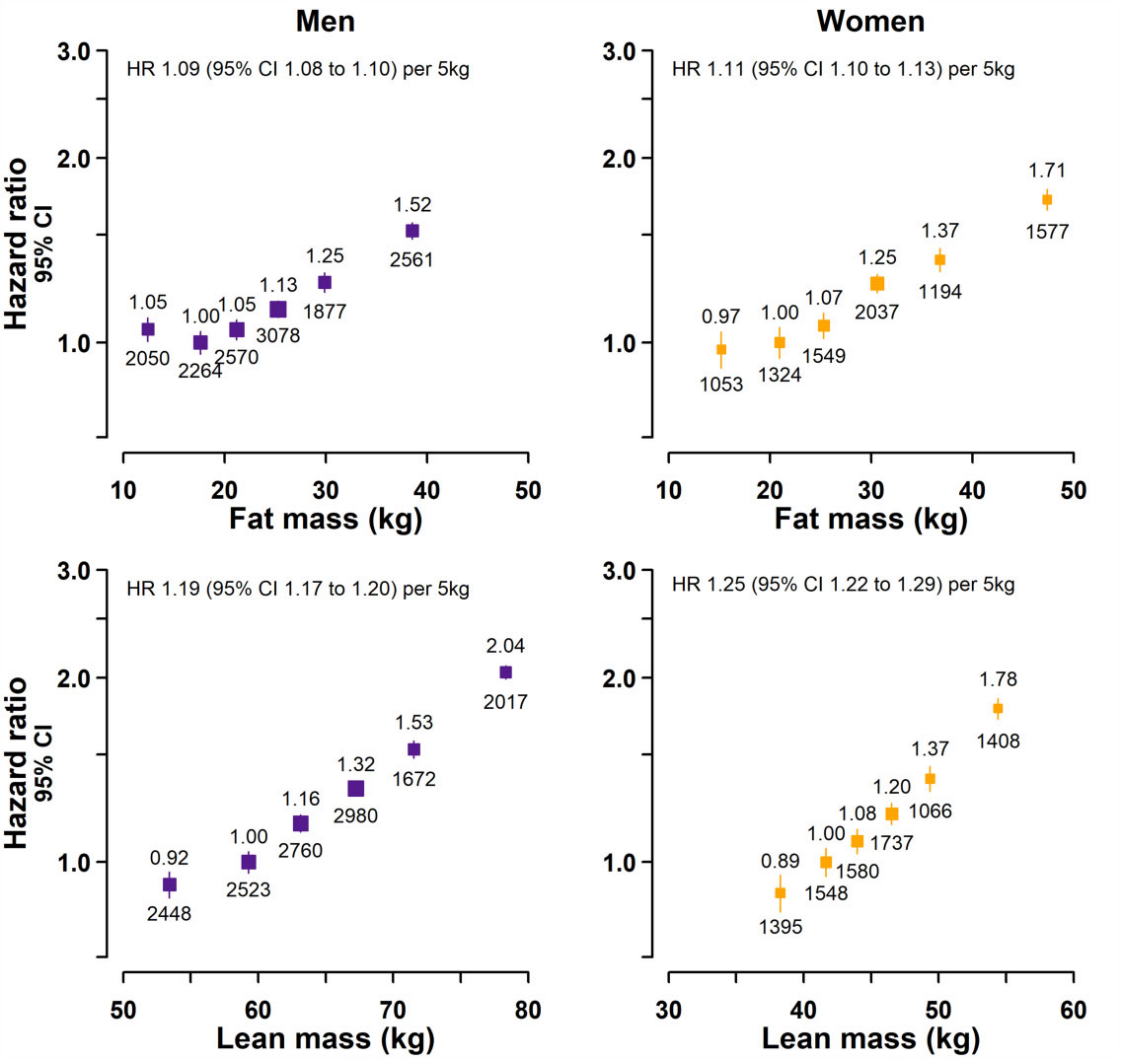
Independent association of lean mass and fat mass with incident atrial fibrillation (AF) by sex. Hazard ratios (HRs) were adjusted for age, ethnicity, deprivation, smoking and alcohol. Associations of fat mass were also adjusted for lean mass, and those of lean mass were also adjusted for fat mass. Exclusions as per Table  1. For each category, the area of the square is inversely proportional to the variance of the category-specific log risk, which also determines the 95% CI (represented by error bars). The lowest four groups each comprise 20% of the sample, with the highest two groups each comprising 10% of the sample. HRs are shown above each square and the numbers of AF cases below. The range of the *x*−axes has been kept consistent between men and women to allow visual comparison.

To assess the independent effects of FM and LM on the risk of AF, we assessed the effect sizes following mutual adjustment. The effect of FM on AF was halved after adjustment for LM (overall and by sex), with a 10% higher risk of AF [HR: 1.10 (1.09–1.11) per 5 kg; Figure  3 and Supplementary Figure S7, available as Supplementary data at *IJE* online]. The effect of LM on AF was attenuated by about one-third after adjustment for FM, showing a 20% higher risk of AF [HR: 1.20 (1.19–1.21) per 5 kg]. However, whilst FM was equally relevant to both sexes, the effect per 5 kg between LM and AF remained slightly weaker in men [HR: 1.19 (1.17–1.20)] than in women [HR: 1.25 (1.22–1.29)], *p*-interaction = 4.3 x 10^−^^9^. These associations were not materially altered by further adjustment for height (Supplementary Figure S8, available as Supplementary data at *IJE* online).

In summary, both FM and LM have independent relevance for risk of AF and overall LM appears to confer about double the risk of AF per kg compared with FM. Although the AF risk associated with FM was similar in men and in women, the association with LM was weaker in men than in women.

### Determining the relative effects of anthropometric measures on risk of AF separately in men and women

**Figure 4:**
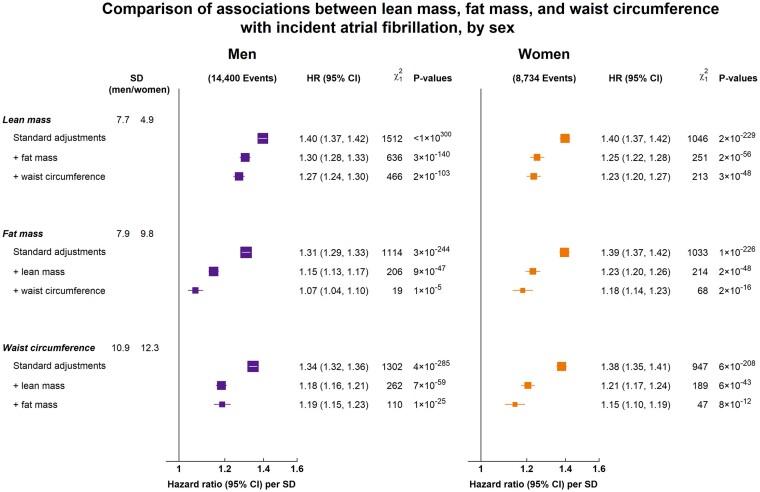
Comparison of associations between lean mass, fat mass, and waist circumference with incident atrial fibrillation, by sex. Hazard ratios (HRs) were adjusted for age, ethnicity, deprivation, smoking and alcohol, with progressive adjustments indicated. Exclusions as per Table  1. For each category, the area of the square is inversely proportional to the variance of the category-specific log risk, which also determines the 95% CI (represented by error bars).

The relative strengths of different body size measures within sex were compared using sex-specific SD units (Supplementary Table S6, available as Supplementary data at *IJE* online). Figure  4 shows the separate effects of LM and FM (as the constituents of weight) and WC (as a measure of central adiposity) on AF, with mutually adjusted effects shown in Supplementary Figure S9 (available as Supplementary data at *IJE* online). In women, the three measures were each independently associated with an ∼20% higher risk per SD (ranging from 15% for WC to 23% for LM). By contrast, in men, LM and WC were associated with a 30% and 20% higher risk per SD, respectively, whereas FM was associated with only an ∼7% higher risk per SD.

As illustrated in Figure  4, LM remained the strongest predictor of AF risk in both sexes after accounting for FM and WC (which together explained 69% of the association in men and 80% in women, based on changes in the χ^2^ statistic). Most of the effect of FM on the risk of AF was explained by LM in both men and women (82% and 79%, respectively). LM also explained much of the effect of WC on the risk of AF (80% in both sexes). As a result, in men, the independent effect of WC was a stronger predictor of AF than FM, whereas, in women, they were more comparable.

### Sensitivity analyses

The impact of regression-dilution bias was assessed based on the subset of participants with data at resurvey (∼4 years after baseline). The mean values of each anthropometric measure were relatively stable over time and regression-dilution ratios were very high (range 0.82–0.94; Supplementary Table S7, available as Supplementary data at *IJE* online). Consequently, correcting for regression-dilution bias did not substantially change the associations, with the exception of those for WC, which became further strengthened (Supplementary Table S6, available as Supplementary data at *IJE* online).

Residual confounding and reverse causation were also considered. The AF associations were comparable following further adjustment for additional potential confounders (physical activity, bread consumption, processed meat consumption, fruit consumption, vegetable consumption; Supplementary Table S8, available as Supplementary data at *IJE* online), for factors potentially on the causal pathway (baseline hypertension and sleep apnoea; Supplementary Table S8, available as Supplementary data at *IJE* online), after censoring those with vascular disease and the first two years of follow-up (Supplementary Table S9, available as Supplementary data at *IJE* online) and within different age groups (Supplementary Figure S10, available as Supplementary data at *IJE* online).

## Discussion

In this large prospective study, incident AF was positively associated with measures of general adiposity, central adiposity and LM. Weight was most strongly associated with the risk of AF with similar effects in both sexes, chiefly driven by the association with LM, albeit LM and FM were both associated with risk of AF. FM had similar effects, per kg, on the risk of AF in both sexes, whereas the effect of LM was weaker in men vs women. Assessment of the relative strength of the associations with different body size measures (LM, FM and WC) suggested different patterns within men and women. In both sexes, LM was the strongest predictor of AF (after mutual adjustment; Figure  4). However, WC was materially stronger than FM in men, whereas this was not the case in women. This sex-related disparity suggests that the relative importance of general and central adiposity in AF prediction may differ between sexes.

Previous prospective studies have reported strong positive associations between BMI and risk of AF.^33–35^ The overall results of the present study are broadly in keeping with those of a previous meta-analysis, which found that 5 kg/m^2^ higher BMI was associated with an ∼30% higher risk of AF.^7^ However, the effects of BMI on the risk of AF in men and women have only been examined in a small number of studies with limited AF cases, resulting in conflicting results; some have suggested a stronger association between BMI and risk of AF in men than in women,^23^^,^^24^ whereas others demonstrated no difference.^36^^,^^37^ In the present large-scale study, there was no difference in the effect of BMI on the AF risk between men and women. In both sexes, following adjustment for WC, the association between BMI and risk of AF was attenuated. Conversely, WC remained strongly predictive of AF after adjustment for BMI. The findings of our study support an independent role for weight and WC, as easy-to-assess clinical measures, in predicting the risk of AF in both sexes and suggest that both should be considered when assessing the risk of an individual developing AF.

Most prospective studies exploring the relationship between adiposity and AF have used BMI as a measure of general adiposity. However, neither BMI nor weight distinguishes between FM and LM or other aspects of body composition, such as body fat distribution, which are important when considering potential differences in association with AF in men and in women. Several studies have used measurements of bio-impedance (or other imaging methods) to estimate FM more directly.^9^^,^^38–41^ A meta-analysis reported a 10% higher risk of AF per 5 kg/m^2^ higher FM.^7^ However, the prospective studies included did not provide comparative strengths of association for men and women, nor did they adjust for the effects of LM.

There is increasing evidence to support an effect of LM on the risk of AF.^23^^,^^33^^,^^34^ Based on an analysis of non-sex-specific SD units among ∼14 000 AF cases in the UKB, Tikkanen *et al.* reported that the effect of LM on the risk of AF was approximately double that of FM (SD = 9.6 kg).^11^ Importantly, LM, FM and measures of central adiposity are highly correlated and adjustment for one or another is required to establish the independent information that these traits contribute to the risk of AF. Fenger-Gron *et al*. in a Danish cohort (3868 AF cases) found that, after adjusting for LM, associations between AF and measures of adiposity were almost completely attenuated.^9^ This finding contrasts with a recent Mendelian-randomization study, which suggests a possible causal role for both FM and LM on the risk of AF.^11^ However, whether there are differences in the effects between men or women has not been established. Our results, based on 23 134 AF cases, suggest some differences between men and women. In women, following adjustment for one or another and WC, the per SD effects of LM and FM on the risk of AF were similar. However, in men, the effect of LM on the risk of AF was greater than that of FM. Differences in risks associated with LM and FM accompanied by a higher contribution of LM to weight in men compared with women may account for some of the observed differences in AF prevalence. LM may vary with age, with lower LM in older adults.^42^ However, our results suggest that LM associations are independent of age, with higher LM associated with greater risk of AF across all age groups studied.

Differences between sexes in the independent effect of FM on the risk of AF have potentially important implications. Although weight loss has been associated with a reduced AF burden,^10^ it has not been clearly established whether this effect is due to a reduction in adipose or lean tissue. In those who are overweight/obese, LM accounts for 20–30% of weight loss due to calorie restriction,^43^ suggesting that LM reduction, which has typically been associated with risk of frailty, may also contribute to the beneficial effects of weight loss on the recurrence rate and burden of AF. Importantly, to date, studies assessing the effect of weight loss on AF have normally combined calorie restriction with physical exercise.^10^^,^^44^ Our findings support public health messaging and individual weight-loss programmes, and suggest that the impact of body composition on the incidence or recurrence of AF, particularly in the context of such programmes, should be further investigated.

The effect of central adiposity on the risk of AF has been less well described than that of general adiposity. Our findings are comparable with a meta-analysis reporting a 1-SD (∼10-cm) higher WC was associated with a 30% higher risk of AF.^8^ However, our study is the first to show a role for central adiposity on the risk of AF independently of LM and general adiposity that is present in both sexes. Consequently, a reduction of 6–15 cm in WC as observed in weight-loss studies, if causal, could translate into an 8–19% and 7–16% lower risk of AF in men and in women, respectively, even after adjustment for LM and FM.^45–47^

WC has been shown to be correlated with visceral abdominal fat in men and women (*r*^2^ = 0.73–0.78).^48^ Visceral adiposity, in particular, has been associated with adverse cardiometabolic risk factors, including elevated blood pressure, dyslipidaemia, inflammatory markers and insulin resistance.^20^^,^^49^^,^^50^ These are, in turn, associated with risk of AF.^1^^,^^19^ Two prospective studies have used body imaging to directly assess the association between abdominal visceral adiposity and the risk of AF, but were insufficiently powered to provide robust conclusions.^39^^,^^51^ Our results suggest that as further abdominal MRI data (including measures of visceral abdominal fat volume) become available, exploration of the relationship between visceral adiposity and AF may improve our understanding of the biological mechanisms by which central adiposity affects the risk of AF.

The effects of adiposity and LM on cardiac structure are not well established. Higher LM has been positively correlated with left ventricular mass and left atrial size,^21^^,^^22^ both of which are associated with risk of AF. In addition, epicardial fat is correlated with a number of anthropometric measures^52^ and may represent a common pathway through which adiposity phenotypes are associated with AF.^8^^,^^53^ Future large-scale data will enable further assessment of these potential mechanisms and help to establish the biological pathways by which LM and measures of adiposity are associated with the risk of AF, which may inform the selection of therapeutic targets.

Our study has a number of strengths, such as its large size and high-quality measurements of adiposity. With >23 000 incident AF events, the study was able to reliably quantify the strengths of the associations and their relative independence, overall and by sex. Furthermore, we were able to adjust for a wide range of potential confounders. Despite this, observational associations can be subject to uncontrolled residual confounding, as well as bias from measurement and correlated errors. Although representativeness is not required for relative risks to be fully generalizable, caution should be used when extending these findings to some population subgroups on which there are limited data (e.g. the young, the very old and some ethnic minority groups). In addition, information on incident AF was taken from hospital records and death registries only; given that AF may initially be diagnosed in the community, there may be a lead-time bias and some participants with asymptomatic paroxysmal AF may not have been identified. We are also unable to assess associations outside of the anthropometric variable ranges observed in the UKB.

## Conclusions

This study quantifies the strengths of the associations between common measures of body composition and incident AF in men and women, and demonstrates a key role of central adiposity and LM in both sexes. However, in contrast with women, the relevance of general adiposity for AF, independently of central adiposity and LM, was more limited in men. Given these findings, the mechanisms by which weight loss leads to improvement in AF in men and women should be further explored.

## Supplementary data

Supplementary data are available at *IJE* online.

## Ethics approval

This research has been conducted using the UK Biobank resource under Application Number 14568. All procedures and data collection in the UK Biobank were approved by the UK Biobank Research Ethics Committee (reference number 11/NW/0274) with participants providing full written informed consent for participation in the UK Biobank and subsequent use of their data for approved applications.

## Funding

This work was supported by the British Heart Foundation [grant numbers FS/20/15/34920 to C.F.C., RG/16/12/3245 and CH/12/3/29609 to B.C. and FS/14/55/30806 to J.C.H.]; the European Union [grant agreement 633196—CATCH ME]; the NIHR; Oxford Biomedical Research Centre; and the Nuffield Department of Population Health, University of Oxford, UK.

## Data availability

UK Biobank data are available in accordance with their published data-access procedures described at http://www.ukbiobank.ac.uk/using-the-resource/.

## Conflict of interest

The Clinical Trial Service Unit and Epidemiological Studies Unit receives research grants from industry that are governed by University of Oxford contracts that protect its independence and has a staff policy of not taking personal payments from industry; further details can be found at https://www.ndph.ox.ac.uk/files/about/ndph-independence-of-research-policy-jun-20.pdf.

## Supplementary Material

dyab184_Supplementary_Data
