# Supplementary material for: Independent effects of adiposity measures on risk of atrial fibrillation in men and women: a study of 0.5 million individuals
Source: Int J Epidemiol. 2021 Sep 25;51(3):984–95. doi: 10.1093/ije/dyab184 (PMC9189979; doi:10.1093/ije/dyab184)
Supplement: dyab184_Supplementary_Data [file dyab184_supplementary_data.zip › ije-2020-12-2335-File007.pdf]

Independent effects of adiposity measures on risk of atrial fibrillation in men and women: A study of 0.5M individuals

Supplementary Methods

Anthropometric measures

Weight was measured using a Tanita BC418MA body composition analyser, and height was measured using a Seca 240 cm height measure with participants standing against a vertical scale in bare feet. Body mass index was calculated as weight (in kg) divided by height (in m) squared. Waist and hip circumference were measured using a Seca 200 cm flexible tape measure. Waist-hip ratio was calculated by dividing waist circumference by hip circumference. Bio-impedance was used to provide data on whole-body fat mass and lean (fat-free) mass, and was measured using a Tanita BC418MA body composition analyser with participants in bare feet.[1] In 2014-15, 5170 participants were resurveyed and underwent dual-energy X-ray absorptiometry scans.

Censor dates

Participants were followed up for incident atrial fibrillation until country-specific censor dates (England: 30th June 2020, Scotland: 31st October 2016, Wales: 29th February 2016).

Sensitivity Analyses

Sensitivity analyses were conducted to (a) evaluate the impact of regression dilution bias by calculating the regression dilution ratio for each anthropometric measure using

the group method (Supplementary Table 6),[2] (b) address residual confounding by further covariate adjustment (i.e. physical activity, bread consumption, processed meat consumption, fruit consumption, vegetable consumption), and (c) consider potential reverse causality by excluding participants with vascular disease prior to baseline and censoring on vascular disease during follow-up, and excluding atrial fibrillation in the first two years of follow-up.

Townsend Deprivation Index

The Townsend deprivation index is a census-based index of deprivation with higher values representing greater levels of deprivation. The index is constructed using four values available from census level data within a geographical region: households without a car, overcrowded households, households not owner-occupied, and persons unemployed.[3] In the UK, values are calculated for each census Output Area.[4] Each participant was assigned a score which corresponded to the census Output Area in which their postcode was located at the time of assessment.[5] Deprivation scores were derived by UK Biobank and are based on the national census data preceding the baseline assessment.

References

1. UK Biobank. *Body Composition Measurement*. 2011 [cited 16th June 2021]; Available from: https://biobank.ndph.ox.ac.uk/ukb/ukb/docs/body_composition.pdf
2. Clarke R, Emberson JR, Breeze E, et al. Biomarkers of inflammation predict both vascular and non-vascular mortality in older men. *Eur Heart J* 2008; **29**: 800-9.
3. Townsend P. Deprivation. *Journal of Social Policy* 2009; **16**: 125-46.
4. Office for National Statistics. *Output Area*. 2016 [cited 9th April 2021]; Available from: https://webarchive.nationalarchives.gov.uk/20160107193025/http://www.ons.gov.uk/ons/guide-method/geography/beginner-s-guide/census/output-area--oas-/index.html
5. UK Biobank. *Data-Field 189: Townsend deprivation index at recruitment*. 2012 [cited 9th April 2021]; Available from: https://biobank.ctsu.ox.ac.uk/crystal/field.cgi?id=189
