# Supplementary material for: Independent effects of adiposity measures on risk of atrial fibrillation in men and women: a study of 0.5 million individuals
Source: Int J Epidemiol. 2021 Sep 25;51(3):984–95. doi: 10.1093/ije/dyab184 (PMC9189979; doi:10.1093/ije/dyab184)
Supplement: dyab184_Supplementary_Data [file dyab184_supplementary_data.zip › ije-2020-12-2335-File008.pdf]

Independent effects of adiposity measures on risk of atrial fibrillation in men and women: A study of 0.5M individuals

Supplementary Tables and Figures

Table of Contents

Table S1: Disease endpoints and codes used for phenotype definitions......2

Table S2: Baseline characteristics in men by body mass index......4

Table S3: Baseline characteristics in women by body mass index......5

Table S4: Partial Pearson's correlation coefficients between bio-impedance and anthropometric measures 6

Table S5: Effect of anthropometric measures on risk of atrial fibrillation in men and women......7

Table S6: Relative ability of different anthropometric measures to predict incident atrial fibrillation......8

Table S7: Regression dilution ratio estimation......9

Table S8: Associations of anthropometric traits with incident atrial fibrillation after sequential covariate adjustment......10

Table S9: Associations of anthropometric traits with incident atrial fibrillation after exclusion of early follow-up and comorbid vascular disease......11

Figure S1......12

Figure S2......13

Figure S3......14

Figure S4......15

Figure S5......16

Figure S6......17

Figure S7......18

Figure S8......19

Figure S9......20

Figure S10......21

Table S1: Disease endpoints and codes used for phenotype definitions

Disease	Code Book	Codes
**Atrial Fibrillation**	ICD10	I48, I480, I481, I482, I483, I484, I489
	OPCS-4	K223, K571, K575, K621, K622, K623, K624, K625, X501, X502
	UKB Code 6	1471, 1483
	UKB Code 5	1524, 1553
**Coronary Heart Disease**		
Myocardial Infarction	ICD10	I21, I210, I211, I212, I213, I214, I215, I216, I217, I218, I219, I22, I220, I221, I222, I223, I224, I225, I226, I227, I228, I229, I23, I230, I231, I232, I233, I234, I235, I236, I237, I238, I239
	UKB Code 6	1075
Angina	ICD10	I20, I200, I201, I202, I203, I204, I205, I206, I207, I208, I209
Other Ischaemic Heart Disease	ICD10	I24, I240, I241, I242, I243, I244, I245, I246, I247, I248, I249, I25, I250, I251, I252, I253, I254, I256, I257, I258, I259
	OPCS-4	K40, K401, K402, K403, K404, K408, K409, K41, K411, K412, K413, K414, K418, K419, K42, K421, K422, K423, K424, K428, K429, K43, K431, K432, K433, K434, K438, K439, K44, K441, K442, K448, K449, K45, K451, K452, K453, K454, K455, K456, K458, K459, K46, K461, K462, K463, K464, K465, K468, K469, K49, K491, K492, K493, K494, K498, K499, K50, K501, K502, K503, K504, K508, K509, K75, K751, K752, K753, K754, K758, K759
	UKB Code 5	1070, 1095, 1523
**Heart Failure**		
Congestive Cardiac Failure	ICD10	I110, I130, I132, I50, I500, I501, I509
	UKB Code 6	1076
Cardiomyopathy [Not HCM]	ICD10	I255, I420, I423, I424, I425, I426, I427, I428, I429, I43, I430, I431, I432, I438
	UKB Code 6	1079
**Other Conditions**		
Valvular Heart Disease	ICD10	I05, I050, I051, I052, I058, I059, I06, I060, I061, I062, I068, I069, I34, I35
	OPCS-4	K25, K251, K252, K253, K254, K255, K258, K259, K26, K261, K262, K263, K264, K265, K268, K269, K27, K271, K272, K273, K274, K275, K276, K278, K279, K28, K281, K282, K283, K284, K285, K288, K289, K29, K291, K292, K293, K294, K295, K296, K297, K298, K299, K30, K301, K302, K303, K304, K305, K308, K309, K318, K319, K328, K329, K341, K342, K343, K344, K345, K346, K348, K349, K356, K357, K358, K359, K361, K362, K368, K369
	UKB Code 6	1078, 1489, 1490, 1584, 1585, 1586, 1587
	UKB Code 5	1097, 1099, 1100, 1101
Stroke	ICD10	I63, I630, I631, I632, I633, I634, I635, I636, I638, I639, I64
	UKB Code 6	1081, 1583
Hypertension	ICD10	I10, I11, I110, I119, I12, I120, I129, I13, I130, I131, I132, I139, I15, I150, I151, I152, I158, I159

Disease	Code Book	Codes
	UKB Code 4	1140851332, 1140851336, 1140851338, 1140851360, 1140851362, 1140851364, 1140851368, 1140851480, 1140851484, 1140851492, 1140851522, 1140851556, 1140851576, 1140851658, 1140851790, 1140851794, 1140851798, 1140851800, 1140860172, 1140860180, 1140860192, 1140860194, 1140860212, 1140860220, 1140860222, 1140860230, 1140860232, 1140860244, 1140860250, 1140860266, 1140860274, 1140860278, 1140860292, 1140860294, 1140860304, 1140860362, 1140860380, 1140860382, 1140860402, 1140860434, 1140860492, 1140860498, 1140860696, 1140860714, 1140860728, 1140860750, 1140860752, 1140860758, 1140860776, 1140860802, 1140860806, 1140860878, 1140860882, 1140860892, 1140860904, 1140860912, 1140860918, 1140861090, 1140861106, 1140861110, 1140861114, 1140861120, 1140861176, 1140861190, 1140861194, 1140861202, 1140861276, 1140861282, 1140863724, 1140864410, 1140866072, 1140866074, 1140866078, 1140866084, 1140866086, 1140866090, 1140866092, 1140866094, 1140866096, 1140866102, 1140866104, 1140866108, 1140866110, 1140866122, 1140866128, 1140866132, 1140866136, 1140866138, 1140866140, 1140866144, 1140866146, 1140866156, 1140866158, 1140866162, 1140866164, 1140866168, 1140866446, 1140866692, 1140866704, 1140866712, 1140866724, 1140866726, 1140866738, 1140866756, 1140866758, 1140866764, 1140866766, 1140866778, 1140866782, 1140866784, 1140866798, 1140866800, 1140866802, 1140866804, 1140872472, 1140872568, 1140875808, 1140875840, 1140878098, 1140879758, 1140879760, 1140879762, 1140879802, 1140879810, 1140879818, 1140879822, 1140879824, 1140879826, 1140879830, 1140879834, 1140879842, 1140879854, 1140879866, 1140881702, 1140881706, 1140881712, 1140881722, 1140881882, 1140881890, 1140888552, 1140888556, 1140888560, 1140888646, 1140888918, 1140888922, 1140909368, 1140909706, 1140910442, 1140910614, 1140911088, 1140916342, 1140916356, 1140916362, 1140916730, 1140916868, 1140916870, 1140916930, 1140917068, 1140917076, 1140922930, 1140923572, 1140923712, 1140923718, 1140926188, 1140926966, 1140927934, 1140927940, 1140928212, 1140928226, 1140928234, 1141145658, 1141145660, 1141145668, 1141145870, 1141146378, 1141150328, 1141150500, 1141150538, 1141150560, 1141151382, 1141152076, 1141152600, 1141152998, 1141153006, 1141153026, 1141153032, 1141156754, 1141156808, 1141156836, 1141156846, 1141157140, 1141162546, 1141164148, 1141164154, 1141164276, 1141164280, 1141166006, 1141166752, 1141167758, 1141167822, 1141168498, 1141169730, 1141170870, 1141171152, 1141171336, 1141171344, 1141172492, 1141172742, 1141173766, 1141179974, 1141182904, 1141182968, 1141184324, 1141187048, 1141187094, 1141187780, 1141187962, 1141188152, 1141188408, 1141188576, 1141188636, 1141188836, 1141188920, 1141188936, 1141190160, 1141190548, 1141193282, 1141193346, 1141194794, 1141199858, 1141199940, 1141200400, 1141200698, 1141200782, 1141201814
	UKB Code 6	1065, 1072
	Touchscreen	High blood pressure reported on touch screen (field 6150) or blood pressure medication reported on touchscreen (field 6177)
	Physical measures	Systolic blood pressure ≥160 mmHg or diastolic blood pressure >100 mmHg
Sleep apnoea	ICD10	G473
	UKB Code 6	1123

ICD= International Disease Classification; HCM=Hypertrophic cardiomyopathy; OPCS-4=Office of Population Censuses and Surveys Classification of Interventions and Procedures Version 4; UKB=UK Biobank. ICD10 and OPCS codes used to determine disease status from HES and death registry data. UKB 4 used to determine medication use at baseline. UKB Code 5 and UKB Code 6 used to determine disease status from verbal interview records.

Table S2: Baseline characteristics in men by body mass index

	BMI Group					
	0-20%	20-40%	40-60%	60-80%	80-90%	90-100%
**Participants**	43,039 (20.0)	43,028 (20.0)	43,061 (20.0)	43,032 (20.0)	21,515 (10.0)	21,521 (10.0)
**Demographic and lifestyle factors**						
Age, mean (SD)	56.0 (8.4)	56.6 (8.3)	56.8 (8.2)	56.9 (8.1)	56.9 (8.0)	56.5 (7.9)
White, n(%)	40,468 (94.0)	40,707 (94.6)	40,827 (94.8)	40,753 (94.7)	20,423 (94.9)	20,559 (95.5)
Current smoker, n(%)	6,784 (15.8)	5,246 (12.2)	4,987 (11.6)	4,981 (11.6)	2,453 (11.4)	2,455 (11.4)
Current alcohol drinker, n(%)	40,168 (93.3)	40,690 (94.6)	40,690 (94.5)	40,535 (94.2)	20,172 (93.8)	19,838 (92.2)
Townsend deprivation index, mean (SD)	-1.2 (3.2)	-1.5 (3.0)	-1.5 (3.0)	-1.3 (3.1)	-1.1 (3.2)	-0.7 (3.3)
**Other anthropometric measures, mean (SD)**						
Height, cm	176.3 (7.0)	175.9 (6.8)	175.6 (6.8)	175.3 (6.7)	175.2 (6.8)	174.9 (6.8)
Weight, kg	71.0 (6.9)	78.9 (6.3)	84.3 (6.7)	90.5 (7.3)	97.7 (7.8)	110.9 (12.3)
Body mass index, kg/m^2	22.8 (1.3)	25.5 (0.6)	27.3 (0.5)	29.4 (0.7)	31.8 (0.6)	36.2 (3.0)
Waist circumference, cm	84.9 (5.9)	91.3 (5.3)	95.8 (5.4)	100.7 (5.7)	106.3 (5.9)	116.3 (8.8)
Hip circumference, cm	96.3 (4.4)	100.1 (4.1)	102.6 (4.2)	105.4 (4.4)	108.8 (4.6)	115.8 (7.5)
Waist-hip ratio,	0.9 (0.1)	0.9 (0.0)	0.9 (0.0)	1.0 (0.1)	1.0 (0.1)	1.0 (0.1)
**Bio-impedance measures, mean (SD)**						
Lean mass, kg	57.4 (5.9)	60.9 (5.7)	63.2 (5.9)	65.7 (6.1)	68.5 (6.4)	73.3 (7.4)
Fat mass, kg	13.5 (3.5)	18.0 (3.2)	21.2 (3.3)	24.9 (3.5)	29.2 (3.7)	37.5 (7.0)
**Prior disease, n(%)**						
Diabetes	1,181 (2.7)	1,531 (3.6)	2,059 (4.8)	3,096 (7.2)	2,225 (10.3)	3,863 (17.9)
Stroke	523 (1.2)	507 (1.2)	609 (1.4)	727 (1.7)	448 (2.1)	561 (2.6)
Coronary heart disease	1,661 (3.9)	2,157 (5.0)	2,626 (6.1)	3,153 (7.3)	1,968 (9.1)	2,359 (11.0)
Congestive cardiac failure	136 (0.3)	147 (0.3)	175 (0.4)	239 (0.6)	181 (0.8)	221 (1.0)
Valvular heart disease	371 (0.9)	358 (0.8)	342 (0.8)	362 (0.8)	207 (1.0)	194 (0.9)

People with missing or out-of-range anthropometric measures, missing covariates, or prior atrial fibrillation at baseline were excluded. BMI – body mass index. *Area level measure of material deprivation (UK range:-5.5 [least deprived] to 14.0 [most deprived]).[31]

Table S3: Baseline characteristics in women by body mass index

	BMI Group					
	0-20%	20-40%	40-60%	60-80%	80-90%	90-100%
**Participants**	52,541 (20.0)	52,524 (20.0)	52,552 (20.0)	52,546 (20.0)	26,273 (10.0)	26,272 (10.0)
**Demographic and lifestyle factors**						
Age, mean (SD)	54.7 (8.2)	56.0 (8.0)	56.8 (7.9)	57.3 (7.8)	57.1 (7.7)	56.3 (7.8)
White, n(%)	50,253 (95.6)	50,255 (95.7)	50,025 (95.2)	49,492 (94.2)	24,504 (93.3)	24,268 (92.4)
Current smoker, n(%)	5,373 (10.2)	4,613 (8.8)	4,566 (8.7)	4,560 (8.7)	2,259 (8.6)	2,098 (8.0)
Current alcohol drinker, n(%)	48,261 (91.9)	48,784 (92.9)	48,323 (92.0)	47,395 (90.2)	23,163 (88.2)	22,428 (85.4)
Townsend deprivation index, mean (SD)	-1.6 (2.9)	-1.7 (2.9)	-1.5 (2.9)	-1.3 (3.0)	-1.0 (3.2)	-0.4 (3.4)
**Other anthropometric measures, mean (SD)**						
Height, cm	163.8 (6.3)	163.0 (6.2)	162.4 (6.2)	161.9 (6.2)	161.5 (6.2)	161.2 (6.3)
Weight, kg	57.0 (5.4)	63.8 (5.1)	69.0 (5.5)	75.7 (6.3)	83.8 (6.8)	98.1 (11.5)
Body mass index, kg/m^2	21.2 (1.3)	24.0 (0.6)	26.1 (0.7)	28.9 (1.0)	32.1 (0.9)	37.7 (3.4)
Waist circumference, cm	71.6 (5.5)	77.7 (5.6)	82.9 (6.1)	89.2 (6.6)	96.1 (7.0)	106.6 (9.3)
Hip circumference, cm	93.1 (4.6)	97.9 (4.3)	101.5 (4.5)	106.3 (5.1)	112.0 (5.6)	122.6 (9.0)
Waist-hip ratio,	0.8 (0.1)	0.8 (0.1)	0.8 (0.1)	0.8 (0.1)	0.9 (0.1)	0.9 (0.1)
**Bio-impedance measures, mean (SD)**						
Lean mass, kg	40.9 (3.4)	42.5 (3.5)	43.7 (3.6)	45.4 (3.8)	47.7 (4.0)	51.9 (5.2)
Fat mass, kg	16.1 (3.6)	21.2 (3.2)	25.3 (3.5)	30.3 (4.0)	36.2 (4.3)	46.2 (7.7)
**Prior disease, n(%)**						
Diabetes	458 (0.9)	643 (1.2)	1,009 (1.9)	1,876 (3.6)	1,676 (6.4)	3,045 (11.6)
Stroke	276 (0.5)	341 (0.6)	446 (0.8)	576 (1.1)	376 (1.4)	429 (1.6)
Coronary heart disease	519 (1.0)	743 (1.4)	1,017 (1.9)	1,435 (2.7)	955 (3.6)	1,222 (4.7)
Congestive cardiac failure	45 (0.1)	49 (0.1)	64 (0.1)	108 (0.2)	74 (0.3)	129 (0.5)
Valvular heart disease	478 (0.9)	467 (0.9)	439 (0.8)	496 (0.9)	246 (0.9)	249 (0.9)

People with missing or out-of-range anthropometric measures, missing covariates, or prior atrial fibrillation at baseline were excluded.
BMI – body mass index. *Area level measure of material deprivation (UK range:-5.5 [least deprived] to 14.0 [most deprived]).[31]

Table S4: Partial Pearson's correlation coefficients between bio-impedance and anthropometric measures

		Lean mass	Fat mass	Body mass index	Waist circumference	Waist-hip ratio	Hip circumference	Height	Weight	Lean mass (DXA)*
**Fat mass**	Male	0.60	1.00							
	Female	0.71	1.00							
	All	0.62	1.00							
**Body mass index**	Male	0.65	0.92	1.00						
	Female	0.69	0.94	1.00						
	All	0.64	0.93	1.00						
**Waist circumference**	Male	0.65	0.89	0.88	1.00					
	Female	0.66	0.88	0.87	1.00					
	All	0.63	0.88	0.87	1.00					
**Waist-hip ratio**	Male	0.32	0.61	0.60	0.80	1.00				
	Female	0.28	0.44	0.46	0.75	1.00				
	All	0.29	0.50	0.51	0.77	1.00				
**Hip circumference**	Male	0.72	0.83	0.81	0.83	0.32	1.00			
	Female	0.72	0.91	0.89	0.82	0.24	1.00			
	All	0.67	0.88	0.86	0.82	0.27	1.00			
**Height**	Male	0.62	0.14	-0.06	0.15	-0.05	0.28	1.00		
	Female	0.48	0.15	-0.12	0.04	-0.08	0.13	1.00		
	All	0.55	0.14	-0.09	0.09	-0.07	0.19	1.00		
**Weight**	Male	0.89	0.90	0.88	0.87	0.53	0.87	0.42	1.00	
	Female	0.86	0.97	0.92	0.86	0.42	0.91	0.28	1.00	
	All	0.86	0.93	0.90	0.86	0.46	0.88	0.34	1.00	
**Lean mass (DXA)***	Male	0.90	0.52	0.55	0.53	0.23	0.64	0.62	0.79	1.00
	Female	0.87	0.60	0.53	0.53	0.22	0.59	0.55	0.75	1.00
	All	0.89	0.54	0.52	0.52	0.22	0.58	0.58	0.76	1.00
**Fat mass (DXA)***	Male	0.59	0.84	0.79	0.79	0.53	0.73	0.19	0.80	0.47
	Female	0.59	0.87	0.83	0.83	0.40	0.79	0.07	0.84	0.53
	All	0.57	0.86	0.82	0.82	0.47	0.76	0.13	0.82	0.49

Correlation coefficients are calculated after adjustment for age at baseline in 5 year bands. Combined sex correlation coefficients are additionally adjusted for sex. DXA = dual-energy X-ray absorptiometry. *Correlations with DXA measures were undertaken in a subset of 4,955 individuals (2,346 men and 2,609 women) with DXA measures of lean mass and fat mass.

Table S5: Effect of anthropometric measures on risk of atrial fibrillation in men and women

	Units	HR (95% CI)	X_1^2
**Men**			
Body mass index	5 kg/m^2	1.39 (1.36 to 1.41)	1102
Waist circumference	10 cm	1.31 (1.29 to 1.33)	1302
Weight	10 kg	1.26 (1.25 to 1.28)	1610
Lean mass	5 kg	1.24 (1.23 to 1.25)	1512
Fat mass	5 kg	1.18 (1.17 to 1.19)	1114
**Women**			
Body mass index	5 kg/m^2	1.34 (1.32 to 1.37)	832
Waist circumference	10 cm	1.30 (1.28 to 1.32)	947
Weight	10 kg	1.29 (1.27 to 1.31)	1187
Lean mass	5 kg	1.40 (1.37 to 1.43)	1046
Fat mass	5 kg	1.18 (1.17 to 1.20)	1033

Hazard ratios (HR) were adjusted for age, sex, ethnicity, deprivation, smoking and alcohol. Effects are reported per the units specified as measured at baseline. χ^2 values derived from type 1 likelihood ratio values.

Table S6: Relative ability of different anthropometric measures to predict incident atrial fibrillation

	HR per 1-SD baseline higher measure		HR per 1-SD usual higher measure	
	Baseline SD	HR (95% CI)	Usual SD	HR (95% CI)
**Men**				
Body mass index, kg/m^2	4.1	1.31 (1.29 to 1.33)	4.0	1.33 (1.31 to 1.35)
Waist circumference, cm	11.0	1.34 (1.32 to 1.36)	10.0	1.43 (1.41 to 1.46)
Weight, kg	13.9	1.38 (1.36 to 1.40)	13.5	1.41 (1.39 to 1.44)
Lean mass, kg	7.7	1.40 (1.37 to 1.42)	7.5	1.43 (1.40 to 1.45)
Fat mass, kg	8.0	1.31 (1.29 to 1.33)	7.6	1.34 (1.32 to 1.36)
**Women**				
Body mass index, kg/m^2	5.0	1.34 (1.32 to 1.37)	4.9	1.37 (1.34 to 1.40)
Waist circumference, cm	12.3	1.38 (1.35 to 1.41)	11.4	1.45 (1.42 to 1.49)
Weight, kg	13.7	1.42 (1.39 to 1.45)	13.2	1.46 (1.43 to 1.49)
Lean mass, kg	4.9	1.40 (1.37 to 1.42)	4.7	1.44 (1.41 to 1.48)
Fat mass, kg	9.8	1.39 (1.37 to 1.42)	9.4	1.44 (1.41 to 1.47)

Hazard ratios (HR) were adjusted for age, sex, ethnicity, deprivation, smoking and alcohol. Effects are reported per sex-specific standard deviation from a measurement at baseline (Baseline SD) and after correction for regression dilution bias (Usual SD).

Table S7: Regression dilution ratio estimation

	Men							Women						
	Means		Standard deviation		Mean range		**Regression dilution ratio**	Means		Standard deviation		Mean range		**Regression dilution ratio**
Adiposity Measure	Baseline	Resurvey	Baseline	Resurvey	Baseline	Resurvey		Baseline	Resurvey	Baseline	Resurvey	Baseline	Resurvey	
**Impedance Measures**														
Lean mass (kg)	63.08	62.03	7.35	7.30	21.11	19.78	**0.94**	44.16	43.55	4.75	4.71	13.23	12.04	**0.91**
Fat mass (kg)	21.34	22.12	7.68	7.84	21.53	19.69	**0.91**	25.93	26.15	9.35	9.39	26.47	24.09	**0.91**
**Anthropomorphic Measures**														
Body mass index (kg/m^2)	27.25	27.33	3.88	3.93	10.95	10.27	**0.94**	26.40	26.45	4.76	4.82	13.56	12.72	**0.94**
Waist Circumference (cm)	95.04	96.56	10.74	10.81	30.52	25.07	**0.82**	82.46	84.51	11.67	12.10	33.69	28.95	**0.86**
Weight (kg)	84.41	84.14	13.32	13.40	37.53	35.42	**0.94**	70.08	69.69	13.04	13.05	36.81	34.33	**0.93**

Baseline and resurvey values provided for participants attending for resurvey between 2009 and 2013 (n=19,283) following the same exclusions as the main analysis. Mean range value is the difference between the mean value within the top 20% (defined by the value at baseline) and mean value within the bottom 20% (defined by the value at baseline). Regression dilution ratio value is derived as the mean range at resurvey divided by the mean range at baseline.

Table S8: Associations of anthropometric traits with incident atrial fibrillation after sequential covariate adjustment

Predictor	Both Sexes			Men Only			Women Only		
	Model A	Model B	Model C	Model A	Model B	Model C	Model A	Model B	Model C
Body mass index, kg/m^2	1.32 (1.31 to 1.34)	1.32 (1.30 to 1.34)	1.25 (1.24 to 1.27)	1.31 (1.29 to 1.33)	1.31 (1.28 to 1.33)	1.24 (1.22 to 1.26)	1.34 (1.32 to 1.37)	1.35 (1.32 to 1.39)	1.26 (1.23 to 1.29)
Waist circumference, cm	1.36 (1.34 to 1.37)	1.36 (1.34 to 1.37)	1.29 (1.27 to 1.25)	1.34 (1.32 to 1.36)	1.34 (1.32 to 1.36)	1.28 (1.26 to 1.30)	1.38 (1.35 to 1.41)	1.39 (1.35 to 1.43)	1.30 (1.27 to 1.32)
Weight, kg	1.40 (1.38 to 1.42)	1.40 (1.38 to 1.42)	1.34 (1.32 to 1.36)	1.38 (1.36 to 1.40)	1.39 (1.36 to 1.41)	1.33 (1.31 to 1.36)	1.42 (1.39 to 1.45)	1.43 (1.40 to 1.47)	1.35 (1.32 to 1.37)
Lean mass, kg	1.40 (1.38 to 1.42)	1.40 (1.38 to 1.42)	1.35 (1.34 to 1.37)	1.40 (1.37 to 1.42)	1.40 (1.38 to 1.42)	1.36 (1.34 to 1.39)	1.40 (1.37 to 1.42)	1.39 (1.36 to 1.42)	1.34 (1.31 to 1.36)
Fat mass, kg	1.34 (1.32 to 1.35)	1.34 (1.32 to 1.35)	1.27 (1.26 to 1.29)	1.31 (1.29 to 1.33)	1.31 (1.29 to 1.33)	1.25 (1.23 to 1.27)	1.39 (1.37 to 1.42)	1.39 (1.36 to 1.42)	1.32 (1.29 to 1.35)

Effects are reported as hazard ratio (95% confidence intervals) per sex-specific standard deviation. Model A: adjusted for age at risk, alcohol status, smoking status, deprivation index. Model B: adjusted for the covariates in Model A + bread consumption, processed meat consumption, fruit and vegetable consumption, and physical activity. Model C: adjusted for the covariates in Model B + baseline hypertension and baseline sleep apnoea

Table S9: Associations of anthropometric traits with incident atrial fibrillation after exclusion of early follow-up and comorbid vascular disease

Anthropometric Measure		No follow-up excluded: No censoring for vascular disease	No follow-up excluded: Vascular disease censored	First two years of follow-up excluded: No censoring for vascular disease	First two years of follow-up excluded: Vascular disease censored
Incident AF Cases	Men	14,400	7,417	12,931	6,627
	Women	8,734	5,372	7,913	4,841
Body mass index	Men	1.31 (1.29 to 1.33)	1.27 (1.24 to 1.30)	1.31 (1.29 to 1.33)	1.28 (1.25 to 1.31)
	Women	1.34 (1.32 to 1.37)	1.33 (1.30 to 1.36)	1.35 (1.32 to 1.37)	1.34 (1.30 to 1.37)
Waist circumference	Men	1.34 (1.32 to 1.36)	1.31 (1.28 to 1.34)	1.35 (1.32 to 1.37)	1.32 (1.29 to 1.35)
	Women	1.38 (1.35 to 1.41)	1.36 (1.33 to 1.40)	1.38 (1.36 to 1.41)	1.37 (1.34 to 1.41)
Weight	Men	1.38 (1.36 to 1.40)	1.40 (1.37 to 1.43)	1.38 (1.36 to 1.41)	1.41 (1.38 to 1.44)
	Women	1.42 (1.39 to 1.45)	1.44 (1.41 to 1.48)	1.42 (1.40 to 1.45)	1.45 (1.42 to 1.49)
Lean mass	Men	1.40 (1.37 to 1.42)	1.46 (1.43 to 1.50)	1.39 (1.37 to 1.42)	1.46 (1.43 to 1.50)
	Women	1.40 (1.37 to 1.42)	1.44 (1.41 to 1.48)	1.40 (1.37 to 1.43)	1.46 (1.38 to 1.45)
Fat mass	Men	1.31 (1.29 to 1.33)	1.29 (1.26 to 1.31)	1.31 (1.29 to 1.33)	1.30 (1.27 to 1.33)
	Women	1.39 (1.37 to 1.42)	1.40 (1.37 to 1.44)	1.40 (1.37 to 1.43)	1.41 (1.38 to 1.45)

Effects are reported as hazard ratio (95% confidence intervals) per sex-specific standard deviation. Cox regression model adjusted for age, alcohol, smoking, ethnicity, and deprivation index. Comorbidities censored – participants with prevalent coronary heart disease, heart failure, stroke, and valvular heart disease were excluded, participants developing incident comorbid events were censored at that point.

Figure S1: Study design

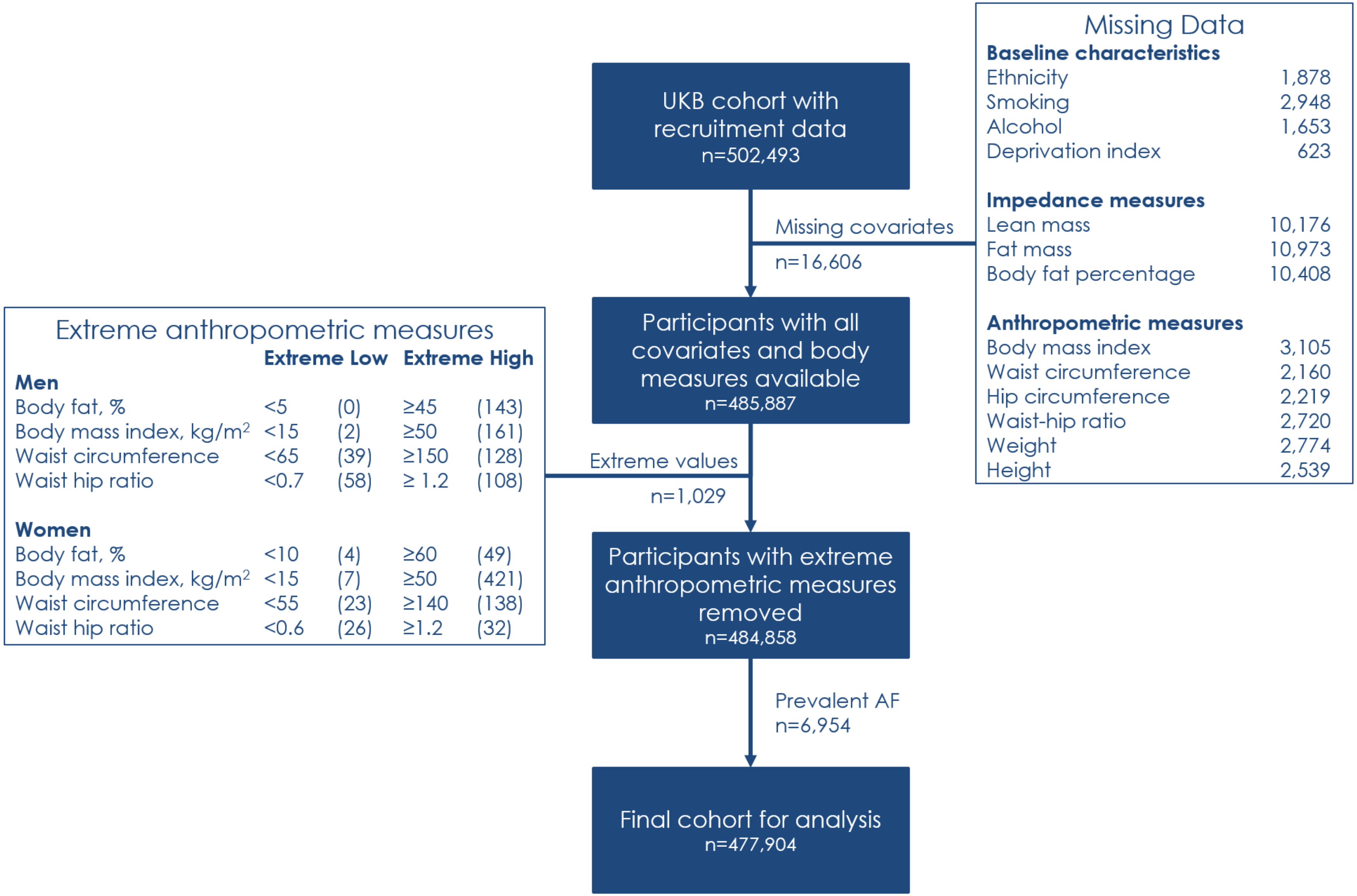

Flow chart illustrating participants included in analyses.

Figure S2: Histograms of anthropometric and bio-impedance variables

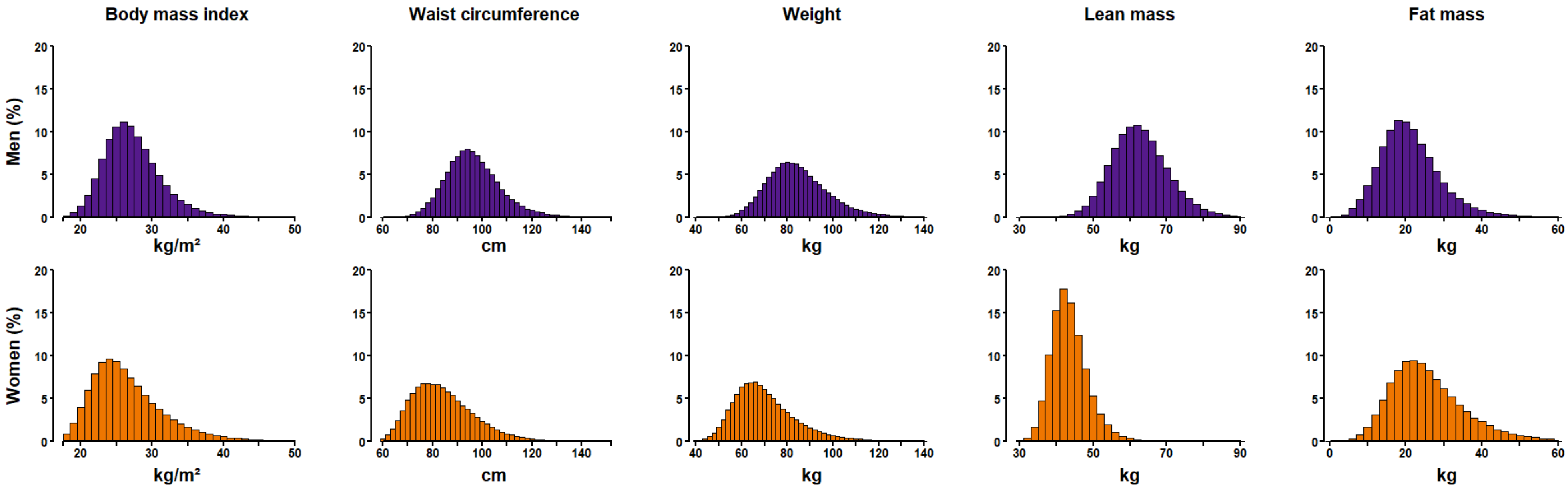

Histograms demonstrating the distribution of values for anthropometric and bio-impedance variables in men and women separately. Exclusions as per table 1.

Figure S3: Incidence of atrial fibrillation (AF) by age

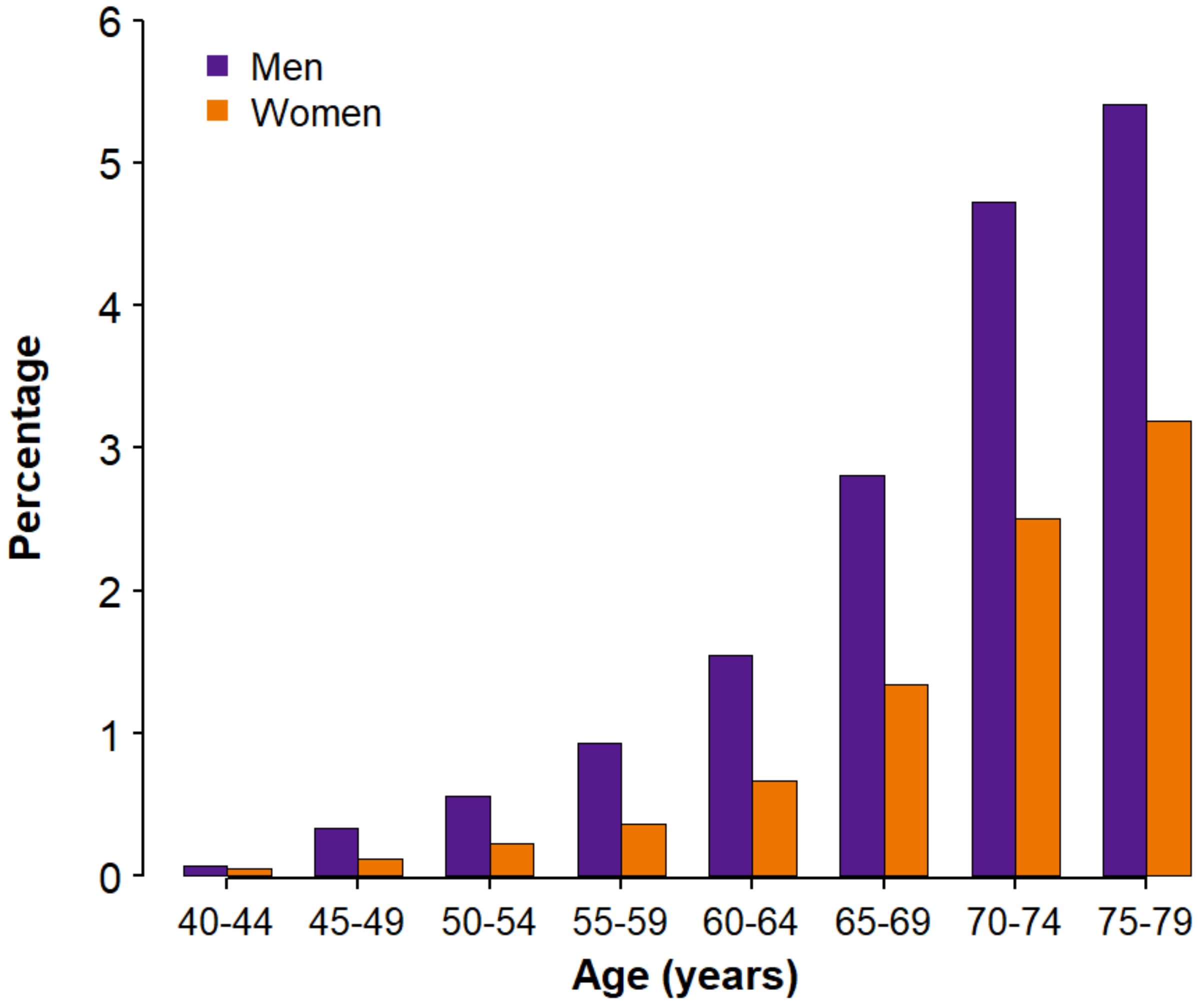

	40-44	45-49	50-54	55-59	60-64	65-69	70-74	75-79
Cases (Men)	17	172	456	955	1929	3777	4508	2419
Cases (Women)	13	77	236	481	1075	2196	2884	1676
Participants (Men)	22966	50937	81566	101870	124904	134469	95509	44754
Participants (Women)	26739	62373	103341	132164	160377	163246	114899	52513

Barplot showing the incidence of AF by age and sex. Exclusions as per table 1. Participants are grouped by age during follow-up. Cases relate to incident AF cases occuring in each age band. Paricipants are those at risk of AF in each age band. Bars represent the percentage developing AF from those at risk of AF within each age group.

Figure S4: Association of body mass index, waist circumference, and weight with incident atrial fibrillation (AF)

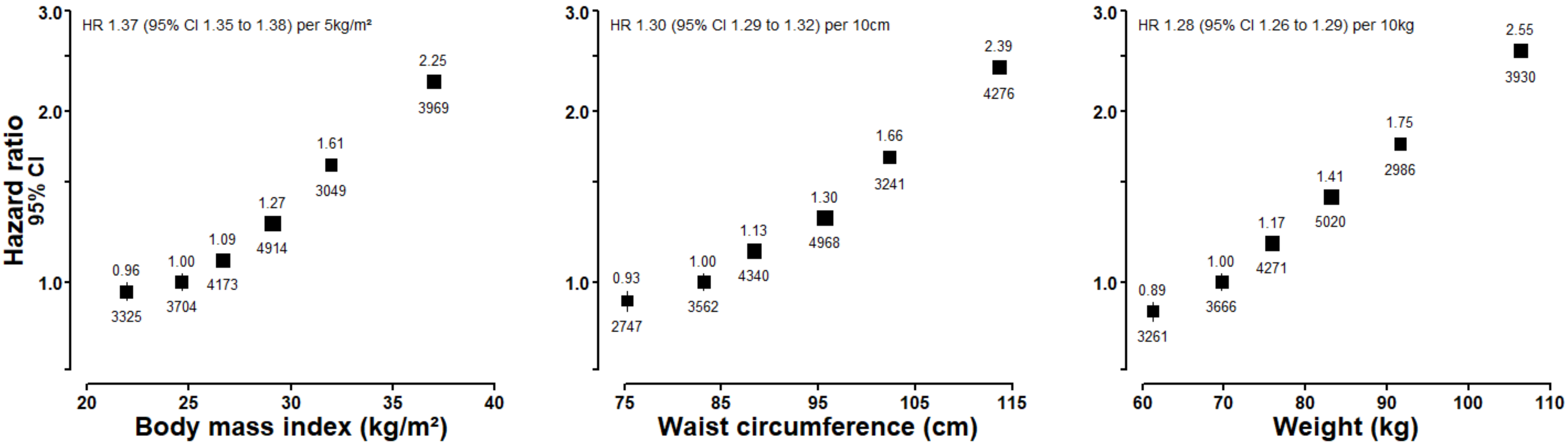

Hazard ratios (HR) were adjusted for age, sex, ethnicity, deprivation, smoking, and alcohol. Exclusions as per table 1. For each category, the area of the square is inversely proportional to the variance of the category-specific log risk, which also determines the 95% CI (represented by error bars). The lowest four groups each comprise 20% of the sample with the highest two groups each comprising 10% of the sample. Hazard ratios shown above each square and the number of AF cases below.

Figure S5: Association of body mass index and weight with incident atrial fibrillation (AF) after adjustment for waist circumference

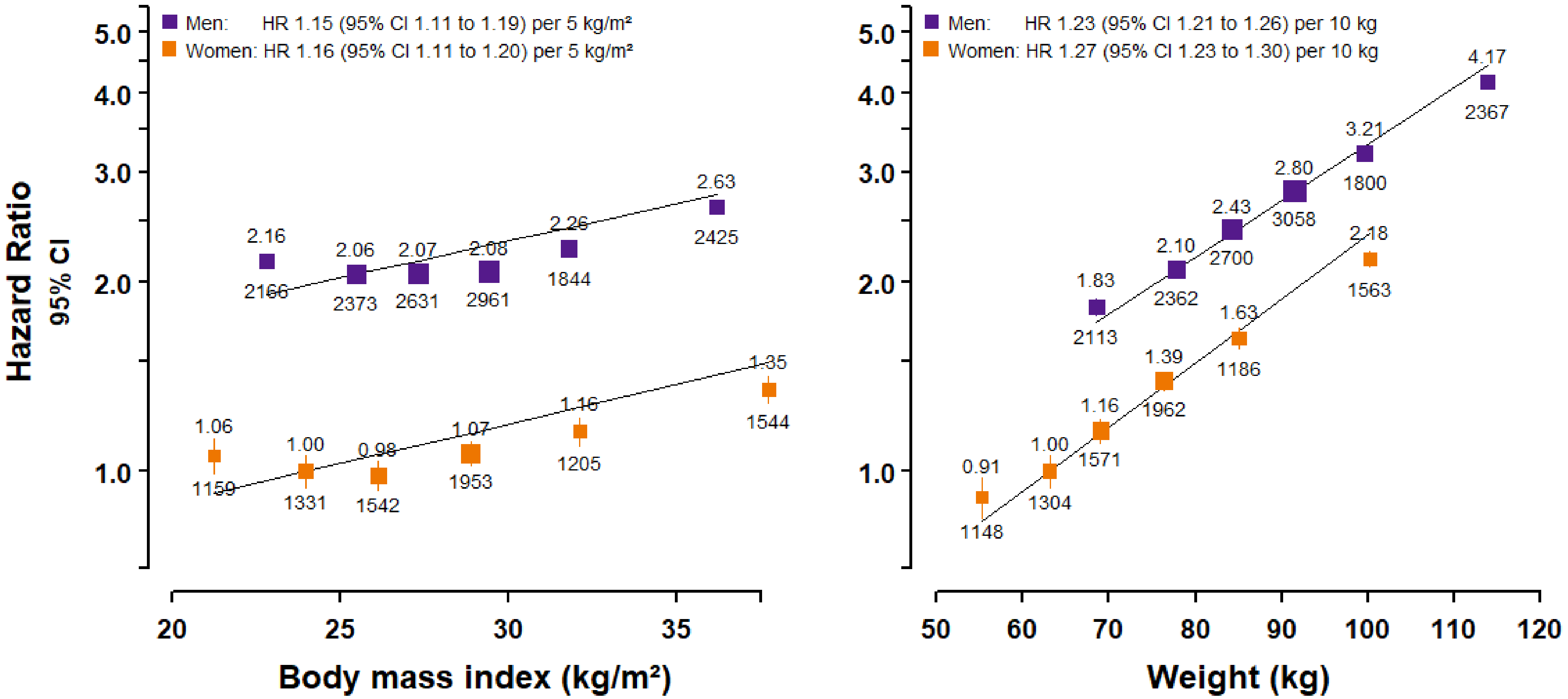

Hazard ratios (HR) were adjusted for age, ethnicity, deprivation, smoking, alcohol, and waist circumference Plotted on a floating absolute scale in men (purple squares) and women (orange squares). Sex-specific regression lines are plotted. Exclusions as per table 1. For each category, the area of the square is inversely proportional to the variance of the category-specific log risk, which also determines the 95% CI (represented by error bars). The lowest four groups each comprise 20% of the sample with the highest two groups each comprise 10% of the sample. Hazard ratios shown above each square and the number of AF cases below.

Figure S6: Association of waist circumference with incident atrial fibrillation (AF) after adjustment for body mass index and weight

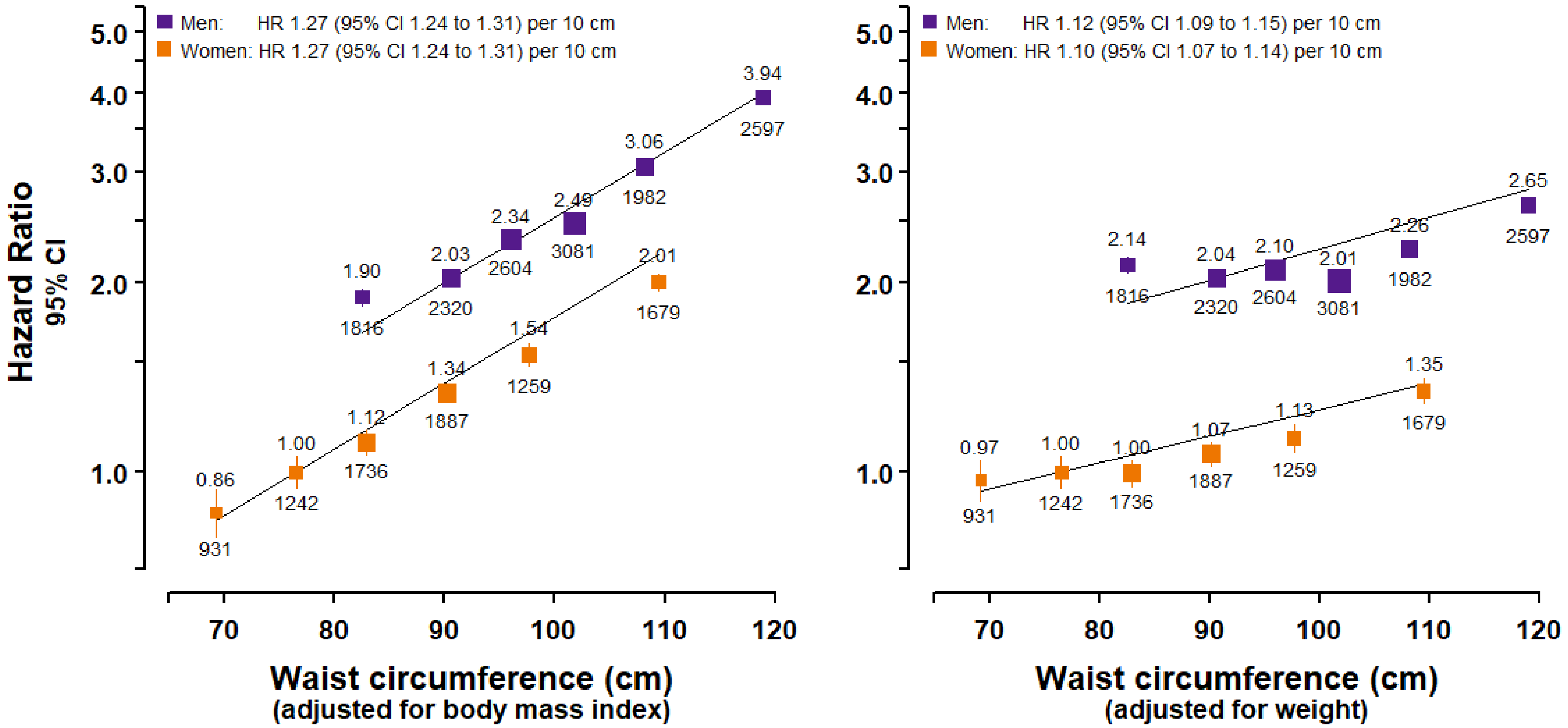

Hazard ratios (HR) were adjusted for age, ethnicity, deprivation, smoking, and alcohol. Further adjustments as detailed in each plot. Plotted on a floating absolute scale in men (purple squares) and women (white squares). Sex-specific regression lines are plotted. Exclusions as per table 1. For each category, the area of the square is inversely proportional to the variance of the category-specific log risk, which also determines the 95% CI (represented by error bars). The lowest four groups each comprise 20% of the sample with the highest two groups each comprise 10% of the sample. Hazard ratios shown above each square and the number of AF cases below.

Figure S7: Association of lean mass and fat mass with incident atrial fibrillation (AF), before and after mutual adjustment

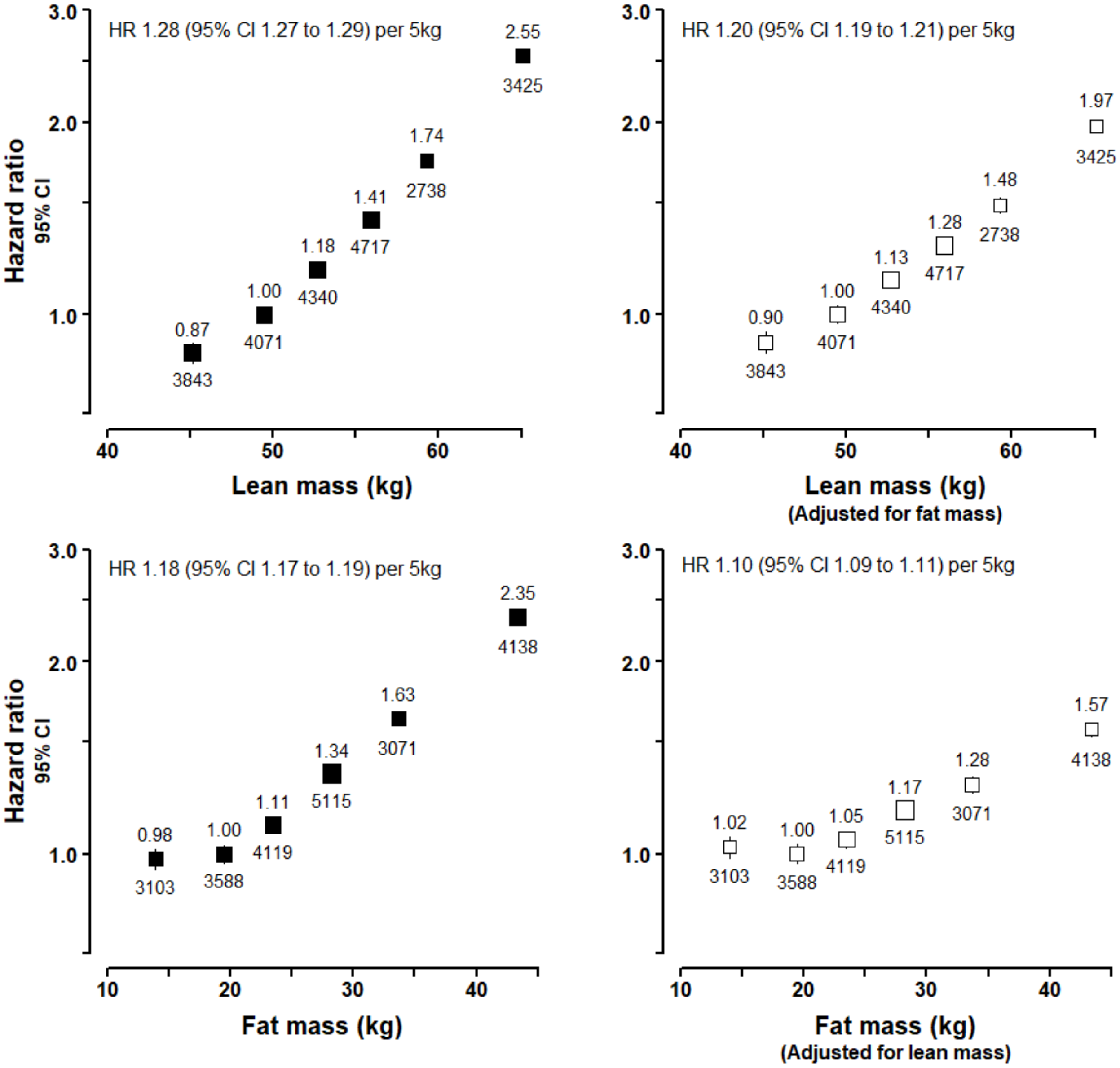

Hazard ratios (HR) were adjusted for age, sex, ethnicity, deprivation, smoking, and alcohol (black squares), with further adjustment as indicated (white squares). Exclusions as per table 1. For each category, the area of the square is inversely proportional to the variance of the category-specific log risk, which also determines the 95% CI (represented by error bars). The lowest four groups each comprise 20% of the sample with the highest two groups each comprising 10% of the sample. Hazard ratios shown above each square and the number of AF cases below.

Figure S8: Association of lean mass and fat mass with incident atrial fibrillation (AF) by sex following mutual adjustment and adjustment for height

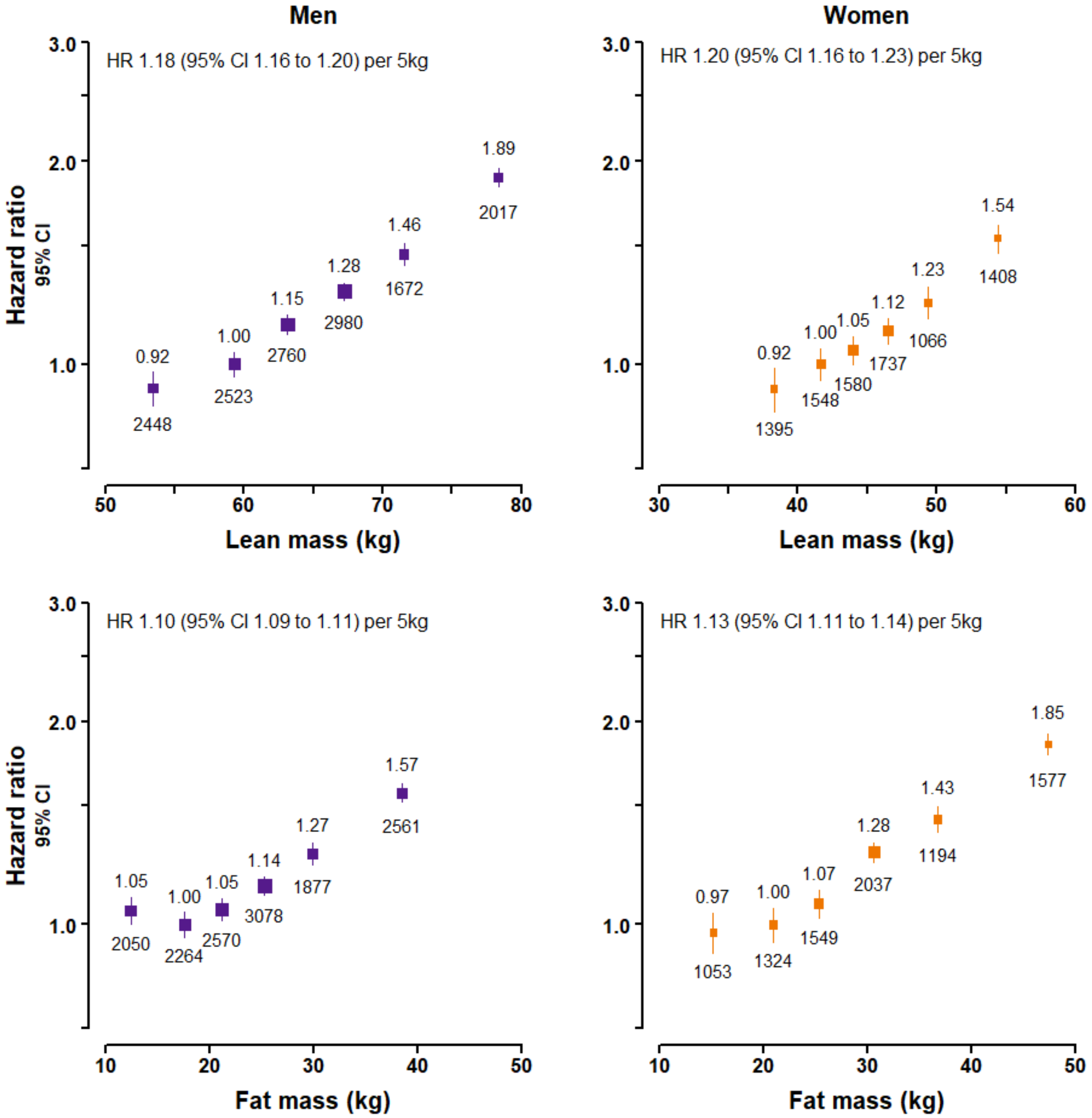

Hazard ratios (HR) were adjusted for age, ethnicity, deprivation, smoking, alcohol, and height. Associations of body fat mass were also adjusted for lean mass, and those of lean mass were also adjusted for body fat mass. Exclusions as per table 1. For each category, the area of the square is inversely proportional to the variance of the category-specific log risk, which also determines the 95% CI (represented by error bars). The lowest four groups each comprise 20% of the sample with the highest two groups each comprising 10% of the sample. Hazard ratios shown above each square and numbers of AF cases below. The range of the X-axes have been kept consistent between men and women to allow for visual comparison.

Figure S9: Association of lean mass, fat mass and waist circumference with incident atrial fibrillation (AF), before and after mutual adjustment

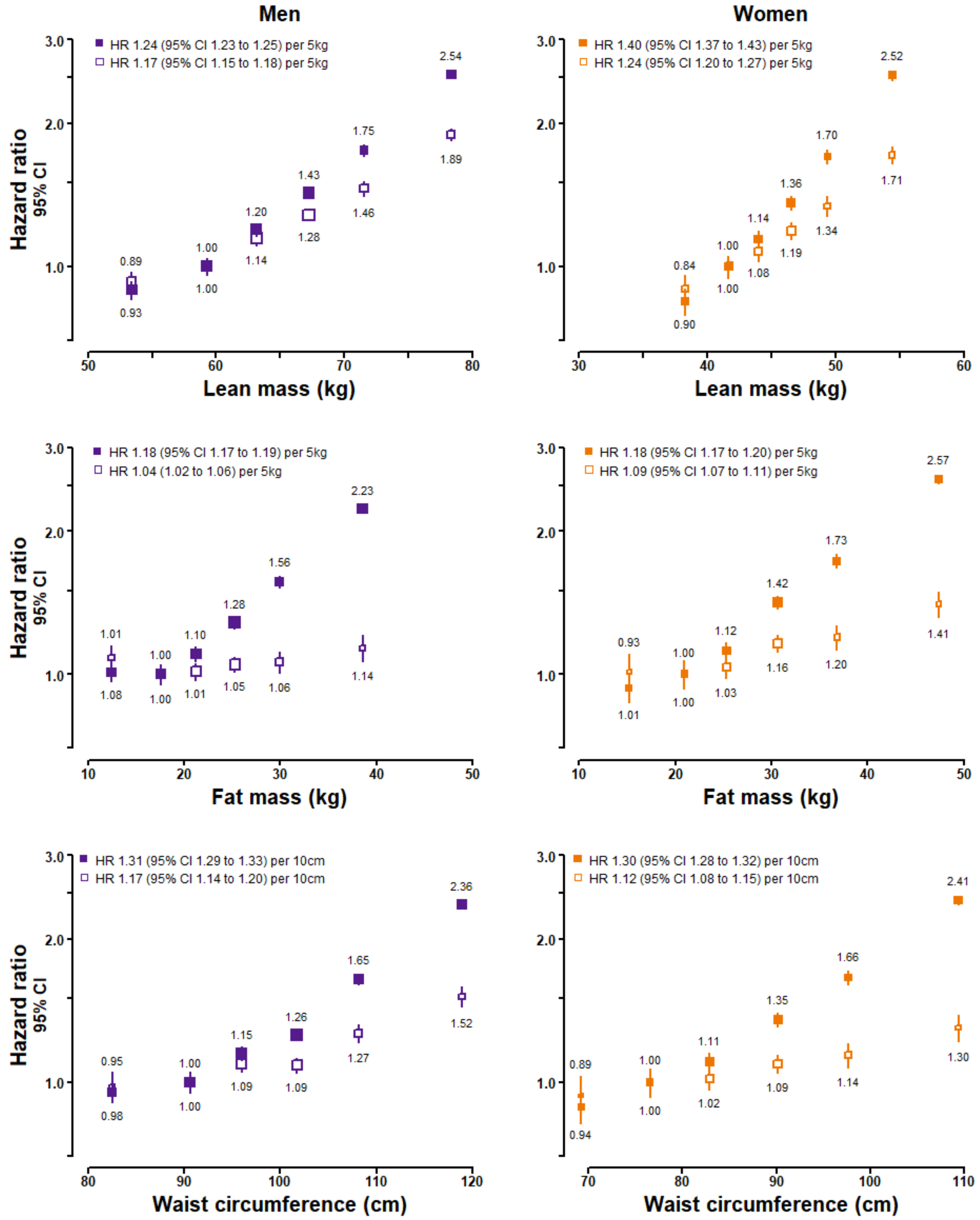

Hazard ratios (HR) were adjusted for age, ethnicity, deprivation, smoking and alcohol (filled squares), with further mutual adjustment (open squares). Exclusions as per table 1. For each category, the area of the square is inversely proportional to the variance of the category-specific log risk, which also determines the 95% CI (represented by error bars). Hazard ratios shown above (filled) and below (open) each square. The lowest four groups each comprise 20% of the sample with the highest two groups each comprising 10% of the sample. The range of the X-axes has been kept consistent between men and women to allow for visual comparison.

Figure S10: Anthropometric measures and risk of atrial fibrillation (AF) by age group

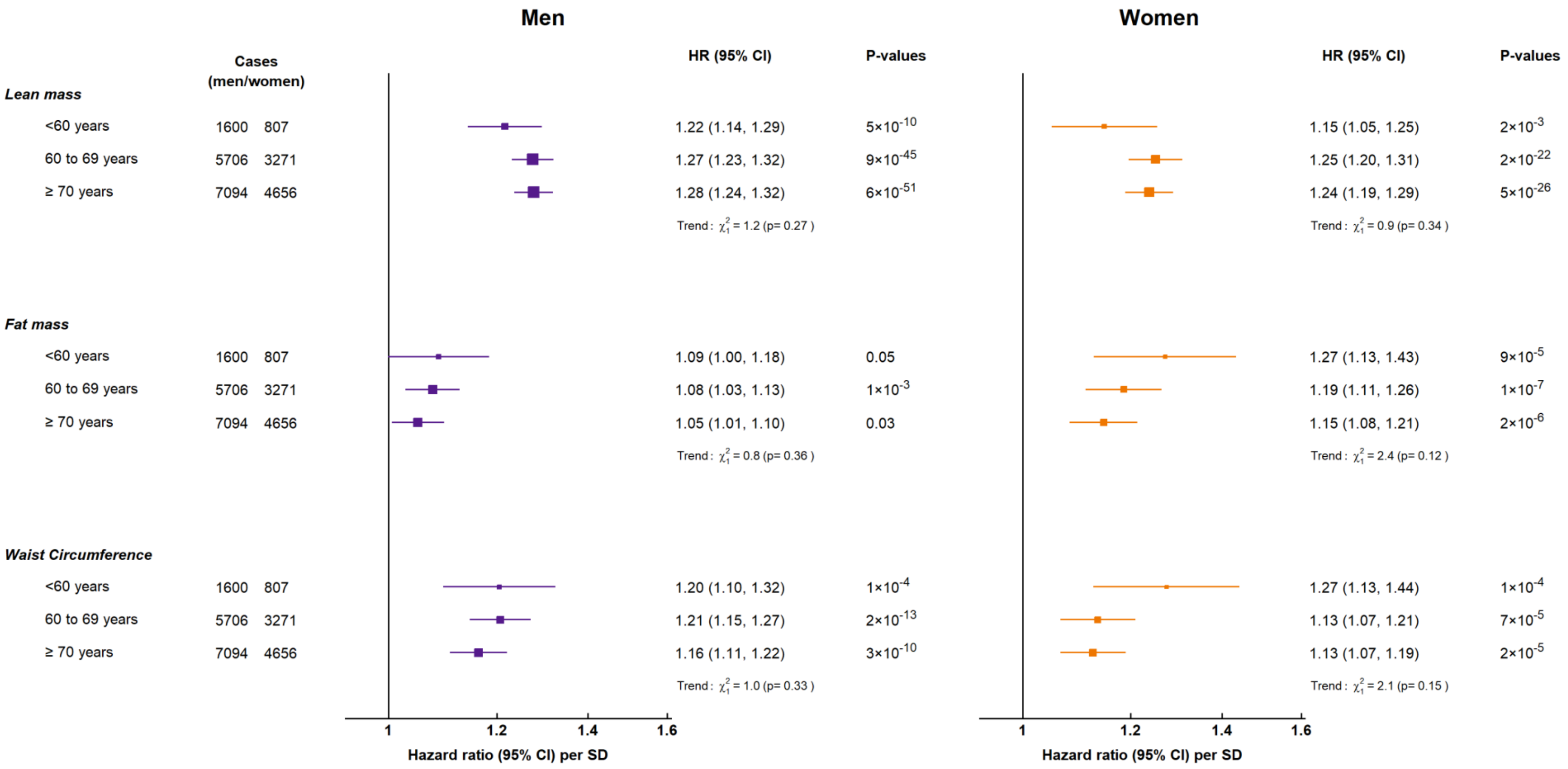

Anthropometric measurs and risk of AF broken down by age group and sex. Exclusions as per table 1. Hazard ratios (HR) were adjusted for age, ethnicity, deprivation, smoking and alcohol. Lean mass additionally adjusted for waist circumference and fat mass. Fat mass additionally adjusted for lean mass and waist circumference. Waist circumference additionally adjusted for fat mass and lean mass. For each category, the area of the square is inversely proportional to the variance of the category-specific log risk, which also determines the 95% CI (represented by error bars).
